# Expression of the Neuregulin Receptor ErbB4 in the Brain of the Rhesus Monkey *(Macaca mulatta)*

**DOI:** 10.1371/journal.pone.0027337

**Published:** 2011-11-08

**Authors:** Jörg Neddens, Andrés Buonanno

**Affiliations:** Section on Molecular Neurobiology, Eunice Kennedy Shriver National Institute of Child Health and Human Development, National Institutes of Health, Bethesda, Maryland, United States of America; University of Salamanca- Medical School, Spain

## Abstract

We demonstrated recently that frontal cortical expression of the Neuregulin (NRG) receptor ErbB4 is restricted to interneurons in rodents, macaques, and humans. However, little is known about protein expression patterns in other areas of the brain. *In situ* hybridization studies have shown high ErbB4 mRNA levels in various subcortical areas, suggesting that ErbB4 is also expressed in cell types other than cortical interneurons. Here, using highly-specific monoclonal antibodies, we provide the first extensive report of ErbB4 protein expression throughout the cerebrum of primates. We show that ErbB4 immunoreactivity is high in association cortices, intermediate in sensory cortices, and relatively low in motor cortices. The overall immunoreactivity in the hippocampal formation is intermediate, but is high in a subset of interneurons. We detected the highest overall immunoreactivity in distinct locations of the ventral hypothalamus, medial habenula, intercalated nuclei of the amygdala and structures of the ventral forebrain, such as the islands of Calleja, olfactory tubercle and ventral pallidum, and medium expression in the reticular thalamic nucleus. While this pattern is generally consistent with ErbB4 mRNA expression data, further investigations are needed to identify the exact cellular and subcellular sources of mRNA and protein expression in these areas. In contrast to *in situ* hybridization in rodents, we detected only low levels of ErbB4-immunoreactivity in mesencephalic dopaminergic nuclei but a diffuse pattern of immunofluorescence that was medium in the dorsal striatum and high in the ventral forebrain, suggesting that most ErbB4 protein in dopaminergic neurons could be transported to axons. We conclude that the NRG-ErbB4 signaling pathway can potentially influence many functional systems throughout the brain of primates, and suggest that major sites of action are areas of the “corticolimbic” network. This interpretation is functionally consistent with the genetic association of *NRG1* and *ERBB4* with schizophrenia.

## Introduction

Single nucleotide polymorphisms (SNPs) in the genes encoding Neuregulin-1 (NRG1) and its receptor ErbB4 are associated with increased risk for and endophenotypes of schizophrenia [1]–[3]; NRG1 is also associated with bipolar disorder [4], [5]. A recent genetic association study reported epistasis between SNPs in *NRG1*, *ERBB4* and *AKT1*, a signaling protein that physically interacts with the cytoplasmic tail of an ErbB4 splice variant known as cyt-1 [6].

The appreciation that ErbB4, initially called Tyro-2 [7], in rodents is expressed in neocortical and hippocampal interneurons [8], [9], and that its expression is restricted to specific subsets of interneurons but is absent from pyramidal cells [10], [11], has been essential for understanding many of the functions of this intricate signaling pathway during neural development and in the adult brain. ErbB4 expression in the primate frontal cortex was found to occur in most interneurons that express either parvalbumin, calretinin or cholecystokinin, but only in the minority of calbindin-positive interneurons [12]. Studies in the frontal cortex and hippocampus of rodents have shown that acute activation of ErbB4 by bath application of NRG1 modulates GABAergic transmission [13]–[15] and synaptic plasticity at glutamatergic synapses [16]–[19]. Moreover, in the hippocampus it has been shown that NRG1 regulates synaptic plasticity at principal neurons by promoting dopamine release, activation of dopamine D4 receptors and subsequent internalization of GluA1-containing AMPA receptors [20]. Furthermore, NRG1-ErbB4 signaling acutely augments hippocampal gamma oscillations [21] and promotes the generation and migration of cortical interneurons [22], [23] as well as the formation and maintenance of interneuron synapses [15], [24]. Taken together, the finding of recruitment of GABAergic, glutamatergic and dopaminergic signaling by the NRG1-ErbB4 pathway provides a biologically plausible mechanism that is consistent with its genetic association (see above) and consolidates many hypotheses on the involvement of different neurotransmitter systems in the etiology of schizophrenia (reviewed in [3]).

However, in order to better understand the possible functional role of this signaling pathway in human disorders, it is imperative to obtain expression data from the brains of primates and humans. Data obtained by *in situ* hybridization indicate ErbB4 transcripts are expressed in many small cells scattered throughout the cerebral cortex of rhesus monkeys and in additional distinct subcortical structures (Allen Brain Atlas [25]). With regard to protein expression, we have recently shown [12] that previous reports on widespread expression of ErbB4 on both interneurons and pyramidal neurons in the cerebral cortex of monkeys and humans [26]–[28] should be taken with caution, because these studies used commercial antibodies of limited specificity. In contrast, using monoclonal antibodies rigorously tested for specificity in tissues of ErbB4 knockout mice, we found that frontal cortical expression of the receptor in monkeys and humans is restricted to the somatodendritic compartment of GABAergic interneurons and is absent from pyramidal cells [12]. These studies showed that the cellular and subcellular distribution of ErbB4 in the frontal cortex is similar across rodents and primates. However, all current expression data are restricted to small brain areas and there is yet no data available on ErbB4 protein expression throughout the entire brain.

Our present study was thus designed to provide a systematic overview on ErbB4 expression throughout major structures of the cerebrum of rhesus monkeys. ErbB4 was detected using highly specific mouse and rabbit monoclonal antibodies that are directed against extracellular and intracellular epitopes, respectively, and microscopic detection and imaging of immunofluorescence were executed in a way that facilitates the comparison of expression levels across different brain sections and brain areas. Our results are presented with a focus on functional systems and indicate that ErbB4 protein expression is considerably higher in “corticolimbic” areas compared to regions that are primarily implicated in sensory and motor functions. In general, our current findings in monkeys, together with previously published data on rodents, suggest that the regional expression pattern of ErbB4 is largely conserved throughout the brains of rodents and primates. These results support the view that studies of the NRG/ErbB4 pathway in rodents can significantly contribute to understanding the etiology and the biological processes altered in psychiatric disorders.

## Materials and Methods

### Ethics Statement

All procedures carried out were in strict adherence to the ILAR “Guide for the Care and Use of Laboratory Animals” and approved by the NIMH Animal Care and Use Committee. The monkey facility is an AAALAC approved facility. Rhesus monkeys were maintained in pairs, under 12-hour dark and light cycles, and were given free access to food and water ad libitum. Three male C57Bl/6J mice (ages p70–p90) were obtained from Jackson Laboratories (Bar Harbour, ME). The animals of this study, rhesus monkeys as well as mice, were not subject to experimental treatment of any kind. Their health was monitored by the attending veterinarian consistent with the recommendations of the Weatherall Report. The animals were euthanized following deep anesthesia, and all efforts were made to minimize suffering.

### Immunohistology

Immunohistological experiments were performed on sections from two male adult rhesus monkeys (*Macaca mulatta*; ages 7 years and 12.5 years) that were kindly provided by Dr. Richard Saunders of the National Institute of Mental Health. Monkeys were deeply anesthetized with sodium pentobarbital and were subsequently euthanized with Euthanasia-iii (Med-Pharmex, Pomona, CA), and then transcardially perfused with 1 L saline followed by 3 L of 4% paraformaldehyde. Heparin (1 ml) was administered i.v. prior to the perfusion. Following perfusion, the extracted brain was cryoprotected in 2 L of 10% glycerol and 2% DMSO in 0.1 M phosphate buffer for 24 h, followed by 2 L of 20% glycerol and 2% DMSO in 0.1 M phosphate buffer for 5 days at 4°C on an orbital shaker. Once the cryoprotection process was complete, the brain was blocked and then frozen using isopentane (4-methyl butane) at −80°C, where it was stored until sectioning. The left hemisphere was sectioned on a sliding microtome using a freezing stage at a thickness of 40 µm. All sections were individually collected in 48-well tissue culture plates, generating ten systematic random sets of ∼130 sections each (total ∼1,300 sections per animal). The sections were stored in cryoprotectant solution (20% glycerol and 30% ethylene glycol in 50 mM phosphate buffer) at −20°C. The perfusion and tissue processing of mice have recently been described in detail elsewhere [12], and the immunofluorescent labeling procedure was identical for monkey and mouse tissues.

Sections were processed for double-immunofluorescence in 0.1 M TBS pH 7.5 with 0.2% Triton-X100 as follows (standard conditions): 2×10 min washes; 1 h in 2% H_2_O_2_ in order to inactivate endogenous peroxidase activity; 3×10 min washes; blocking of endogenous streptavidin-biotin binding sites according to the manufacturer's instructions (Vector Laboratories, Burlingame, CA); 3×10 min washes; 1 h blocking in 10% normal donkey serum; incubation in primary antibodies for 40 h at 4°C in blocking solution; 3×10 min washes; biotinylated and/or fluorophor-labeled secondary antibodies for 1 h in blocking solution; 3×10 min washes; streptavidin-peroxidase (Sigma Aldrich, St. Louis, MO) for 1 h; 3×10 min washes. Sections were then stained with Tyramide-Alexa 488 (Invitrogen, Carlsbad, CA) according to the manufacturer's instructions, washed 3×10 min, mounted in Mowiol-DABCO, and stored at 4°C. All steps of the protocol were executed on an orbital shaker at 50 rpm and were done at room temperature unless stated otherwise. Minimum solution volumes per brain section were as follows: washes in 5 ml buffer (mice: 1 ml), incubation in antibodies, avidin or tyramide in 2 ml (mice: 0.4 ml); the smaller monkey sections of the prefrontal cortex and the nucleus accumbens were incubated together, using the same volume that was used for the larger sections through the amygdala or the hippocampus.

In order to reveal intracellular ErbB4 immunoreactivity, heat-induced antigen retrieval with buffer U at 121°C was used according to the manufacturer's instructions (2100 Retriever, PickCell Laboratories, Amsterdam, Netherlands). Following antigen retrieval, the standard protocol was followed as described above. As controls, sections without primary and/or secondary antibodies and/or streptavidin exhibited only low-intensity general background fluorescence and autofluorescent grains; an example obtained from the anterior cingulate cortex is provided. Control experiments were performed in parallel with other experiments using the same solutions on monkey and mouse sections with and without antigen retrieval. Primary antibodies are shown in Table 1. Secondary antibodies: donkey anti-rabbit Cy3 (1∶1000), donkey anti-mouse Cy3 (1∶1000), donkey anti-goat Cy3 (1∶1000), donkey anti-rat Cy 3(1∶500), donkey anti-mouse Cy2 (1∶300), biotinylated donkey anti-rabbit (1∶1000) and biotinylated donkey anti-mouse (1∶500; all from Jackson ImmunoResearch, West Grove, PA). All secondary antibodies were highly affinity-purified (ML quality) to minimize cross-reactivity.

**Table 1:** List of primary antibodies.

Species	Antigen	Clone	Source	Item #	Dilution
mouse	parvalbumin	PARV-19	Sigma-Aldrich, St. Louis, MO	P3088	1∶5000
rabbit	parvalbumin	poly	Swant, Bellinzona, CH	PV-25	1∶1500
rabbit	nNOS	poly	Invitrogen, Carlsbad, CA	61–7000	1∶4000
rabbit	calretinin	poly	LabVision, Fremont, CA	RB-9002-P0	1∶ 2000
mouse	ErbB4, mAb-77	H4.77.16	LabVision	MS-270	3 µg/ml
rabbit	ErbB4, mAb-10	mAb-10	Vullhorst et al., 2009	mAb-10	3 µg/ml
rabbit	neurogranin	poly	Millipore, Temecula, CA	AB5620	1∶2000
mouse	GAD67	1G10.2	Millipore	MAB5406	1∶5000
rabbit	GAD67	poly	Millipore	AB9706	1∶2000
rat	somatostatin	YC7	Millipore	MAB354	1∶200
goat	ChAT	poly	Millipore	AB144P	1∶500
rabbit	tyrosine hydroxylase	poly	Novus, Littleton, CO	NB300-109	1∶2000
mouse	neurofilament H	SMI-32	Abcam, Cambridge, UK	ab75475	1∶2000

Abbreviations: choline acetyltransferase (ChAT), glutamic acid decarboxylase 67 kD (GAD67), neuronal nitric oxide synthase (nNOS).

The rabbit monoclonal anti-ErbB4 antibody directed against the intracellular domain (mAb-10) has undergone stringent tests for specificity in a variety of applications that include Western blotting, immunoprecipitation, and immunolabeling of cultured hippocampal neurons and brain sections [11]. The specificity of the mouse monoclonal mAb-77 directed against the natively folded extracellular domain of human ErbB4 was demonstrated previously [11], [29]. Moreover, specificity of mAb-10 and mAb-77 was demonstrated on monkey and human brain tissue [12]. Major neuronal cell classes were identified using selective and specific antibodies against neurogranin, neurofilament H and GAD67. Neurogranin is a selectively marker for the majority of glutamatergic principal neurons in the telencephalon of both rodents and primates [30], [31]. Neurofilament H was detected with a widely used and well characterized monoclonal antibody; the protein is expressed in principal neurons projecting to distant areas, and the amount of immunoreactivity positively correlates with the size of the cells [32]. GAD67 is selectively expressed by GABAergic neurons and is a marker for interneurons in the cerebral cortex; however, immunoreactivity may be absent from certain subpopulations of interneurons [33]. Selectivity and specificity of the GAD67 antibody was tested by co-immunofluorescence with several other markers of interneurons on rodent and monkey brain sections, including GABA, and a GAD67 antibody that is directed against a different epitope.

### Microscopy and imaging

Immunofluorescence was analyzed using a confocal microscope (Zeiss 510 Meta) at 10x, 20x, 40x Oil and 63x Oil magnification. Low magnification images were obtained in a single z-plane, whereas higher magnification images (40x and 63x) represent maximum projections of image stacks of 4–8 z-planes. The separation of image planes along the z-axis was set using the “Optimal Interval” function within the Zeiss LSM 5 acquisition software that automatically considers the objective in use and additional microscope settings; the usual separation of z-planes was in the range of 0.3–0.5 µm with a 20% overlap of subsequent z-planes. Large image arrays of entire sections were obtained with a fully motorized Zeiss AxioImager Z1 epifluorescence microscope at 10x magnification in a single z-plane, and automatically collected and assembled from up to 1100 single images with Zeiss AxioVision 4.8 software. The presented images are representative for the staining patterns, and were adjusted for overall brightness and contrast using Adobe Photoshop CS4.

**Figure 1:**
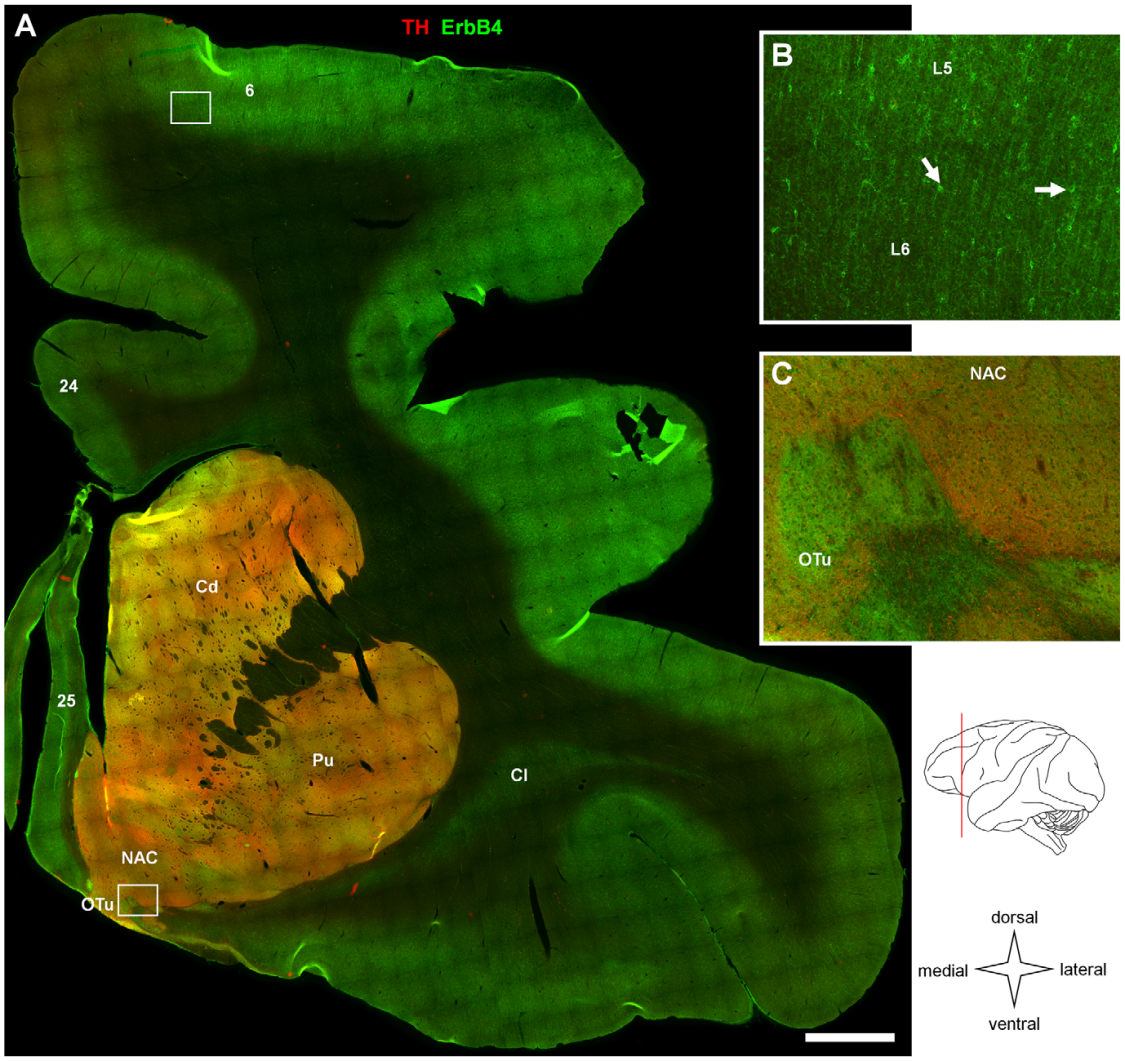
Overview on ErbB4 expression in nucleus accumbens, dorsal striatum and surrounding cortex. (A) Single epifluorescence images (868) of a frontal lobe section double-labeled for ErbB4 (mAb-77, green channel) and TH (red channel) were taken at 10x magnification and automatically assembled. The drawing indicates the approximate anteroposterior position of the section (red line). Immunofluorescence of TH highlights the Cd, Pu, NAC and OTu. ErbB4 expression is evident in all cortical areas; however, immunoreactivity is stronger in dorsal and lateral cortex compared to medial and ventral areas. ErbB4 is expressed at medium levels in the striatum with patches of higher immunofluorescence being evident in the NAC and OTu. The location of areas of interest shown at higher magnification in panels B and C is indicated by rectangles. (B) ErbB4 expression is restricted to small multipolar neurons (arrows) in deep layers of cortical area 6, whereas large, fuzzy areas of high intensity (C) occur at the border of OTu and NAC in the ventral forebrain. Scale bar = 2 mm (A), 220 µm (B, C). Abbreviations: Supplementary motor area and premotor cortex (6), anterior cingulate cortex (24), subgenual prefrontal cortex (25), caudate (Cd), claustrum (Cl), cortical layers (L5, L6), nucleus accumbens (NAC), olfactory tubercle (OTu), putamen (Pu).

**Figure 2:**
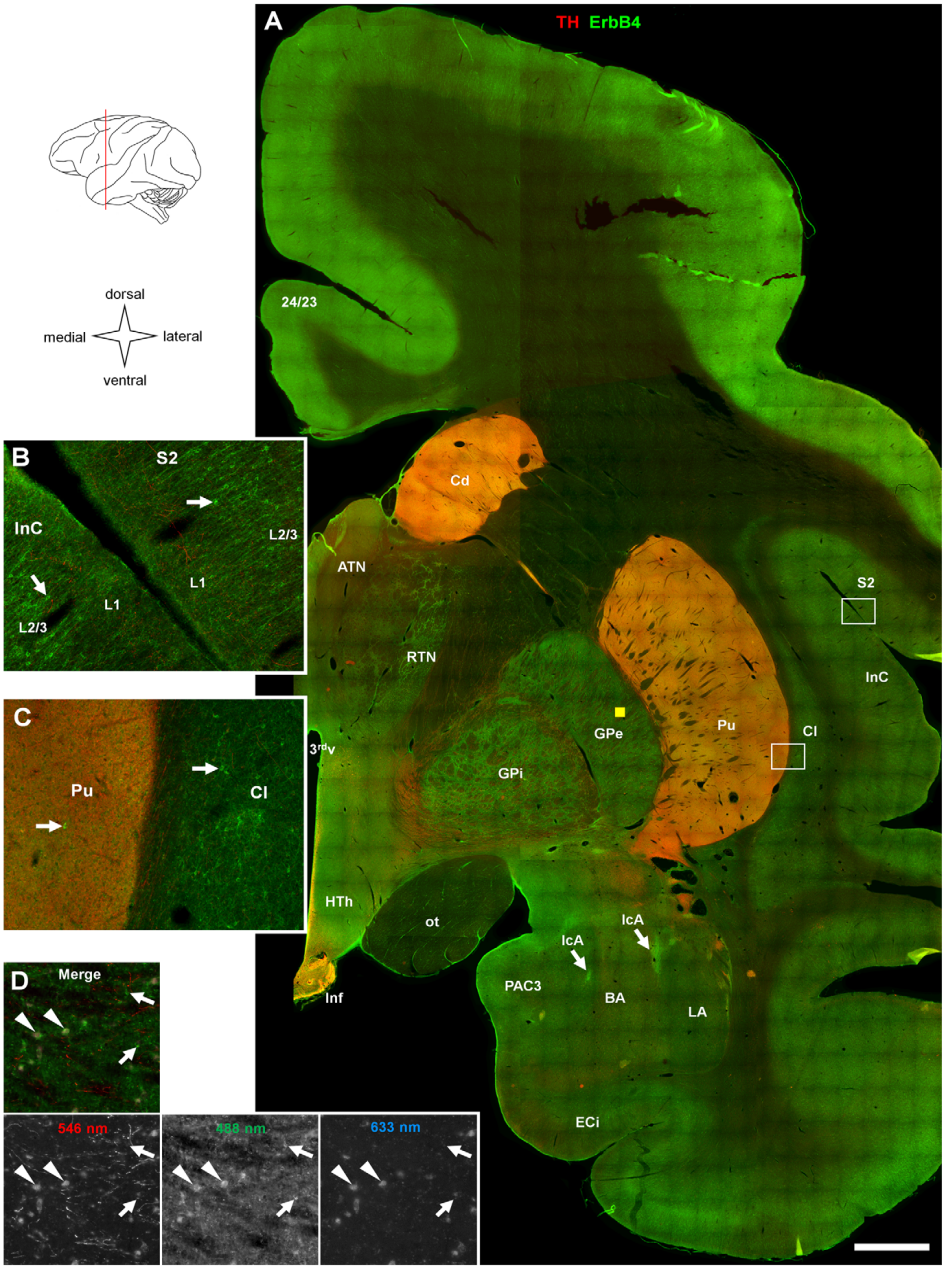
Overview on ErbB4 expression in dorsal striatum, pallidum, thalamus, hypothalamus, amygdala, and surrounding cortex. (A) Single epifluorescence images (1064) were taken at 10x magnification and automatically assembled; antibody staining and channels as in Figure 1. The drawing indicates the approximate anteroposterior position of the section (red line). Immunofluorescence of TH highlights Cd, Pu, and Inf. ErbB4 expression is evident in all cortical areas; however, immunoreactivity is stronger in dorsal compared to ventral areas. ErbB4 is expressed at high levels in patches in the amygdala that correspond to the intercalated nuclei (arrows in A). High concentration of ErbB4 is also evident in the ventral HTh and Inf. Original resolution is shown in apical layers of cortical areas S2 and InC (B) and in the basal ganglia at the border of Pu and Cl (C); ErbB4 expression (green channel) is evident in small multipolar neurons in these areas (arrows). In addition, the Pu shows a medium level of fuzzy immunofluorescence that is absent from the Cl. The position of (B, C) is indicated by rectangles in (A). (D) Fluorescent imaging control in GPe (location is indicated by the yellow box in A) distinguishes autofluorescence (indicated by arrowheads) from specific immunoreactivity in the red and green channels (arrows); note signal on somata in the far red channel (633 nm) that reveals unspecific fluorescence (see Methods for more information). Scale bar = 2 mm (A), 220 µm (B, C), 160 µm (D). Abbreviations: third ventricle (3^rd^v), anterior/posterior cingulate cortex (24/23), anterior thalamic nucleus (ATN), basal amygdala (BA), caudate (Cd), claustrum (Cl), cortical layers (L1, L2/3), internal entorhinal cortex (ECi), globus pallidus externa/interna (GPe/i), hypothalamus (HTh), intercalated amygdala (IcA), infundibulum (Inf), insular cortex (InC), lateral amygdala (LA), optic tract (ot), periamygdaloid cortex 3 (PAC3), putamen (Pu), reticular thalamic nucleus (RTN), somatosensory cortex (S2).

**Figure 3:**
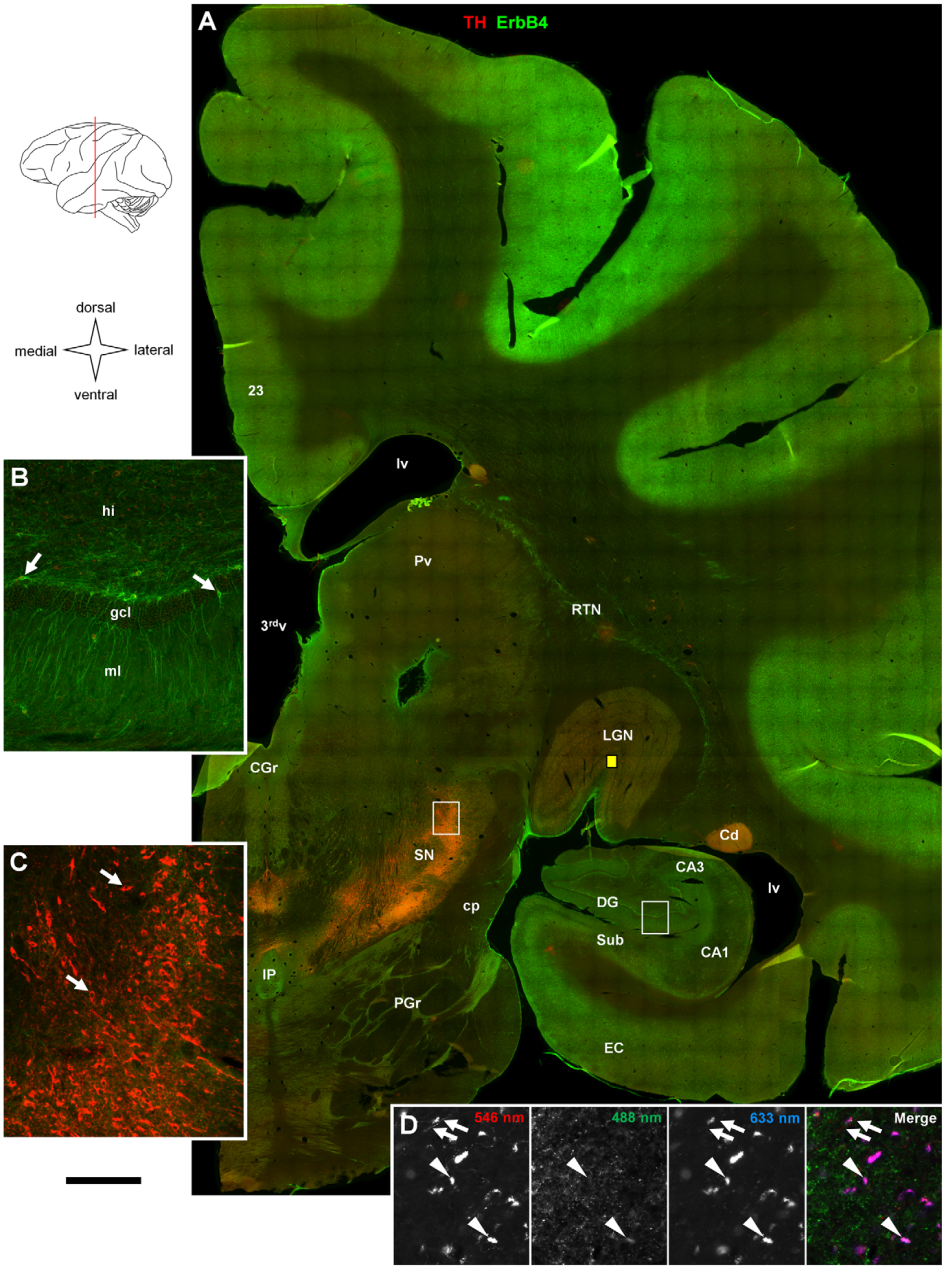
Overview on ErbB4 expression in caudal thalamus, hippocampus, surrounding cortex, mesencephalon, and metencephalon. (A) Single epifluorescence images (1102) were taken at 10x magnification and automatically assembled; antibodies and channels as in Figure 1. The drawing indicates the approximate anteroposterior position of the section (red line). ErbB4 expression is evident in all cortical areas; however, immunoreactivity is stronger in dorsal compared to ventral areas. Several brainstem nuclei (CGr, IP, lateral PGr) show medium to high intensity immunofluorescence. Original resolution is shown in hippocampal dentate gyrus (B) where ErbB4 expression is restricted to small multipolar neurons (arrows), and in the substantia nigra (C) where immunofluorescence in the green channel is generally weak compared to the cortex. Immunofluorescence of TH highlights Cd and somata in the dopaminergic brainstem nuclei (arrows in C). (D) Please note that red signal in the LGN (location is indicated by the yellow box in A) derives mostly from autofluorescence (indicated by arrowheads in 546 nm and 633 nm images) rather than from specific immunoreactivity; arrows identify a TH-positive axon in the 546 nm image. Scale bar = 2 mm (A), 220 µm (B–D). Abbreviations: Third ventricle (3^rd^v), posterior cingulate cortex (23), cornu ammonis 1/3 (CA1/3), caudate (Cd), central gray (CGr), cerebral peduncle (cp), dentate gyrus (DG), entorhinal cortex (EC), granule cell layer (gcl), hilus (hi), interpeduncular nucleus (IP), lateral geniculate nucleus (LGN), lateral ventricle (lv), molecular layer (ml), pontine gray (PGr), pulvinar (Pv), reticular thalamic nucleus (RTN), subiculum (Sub), substantia nigra (SN).

**Figure 4:**
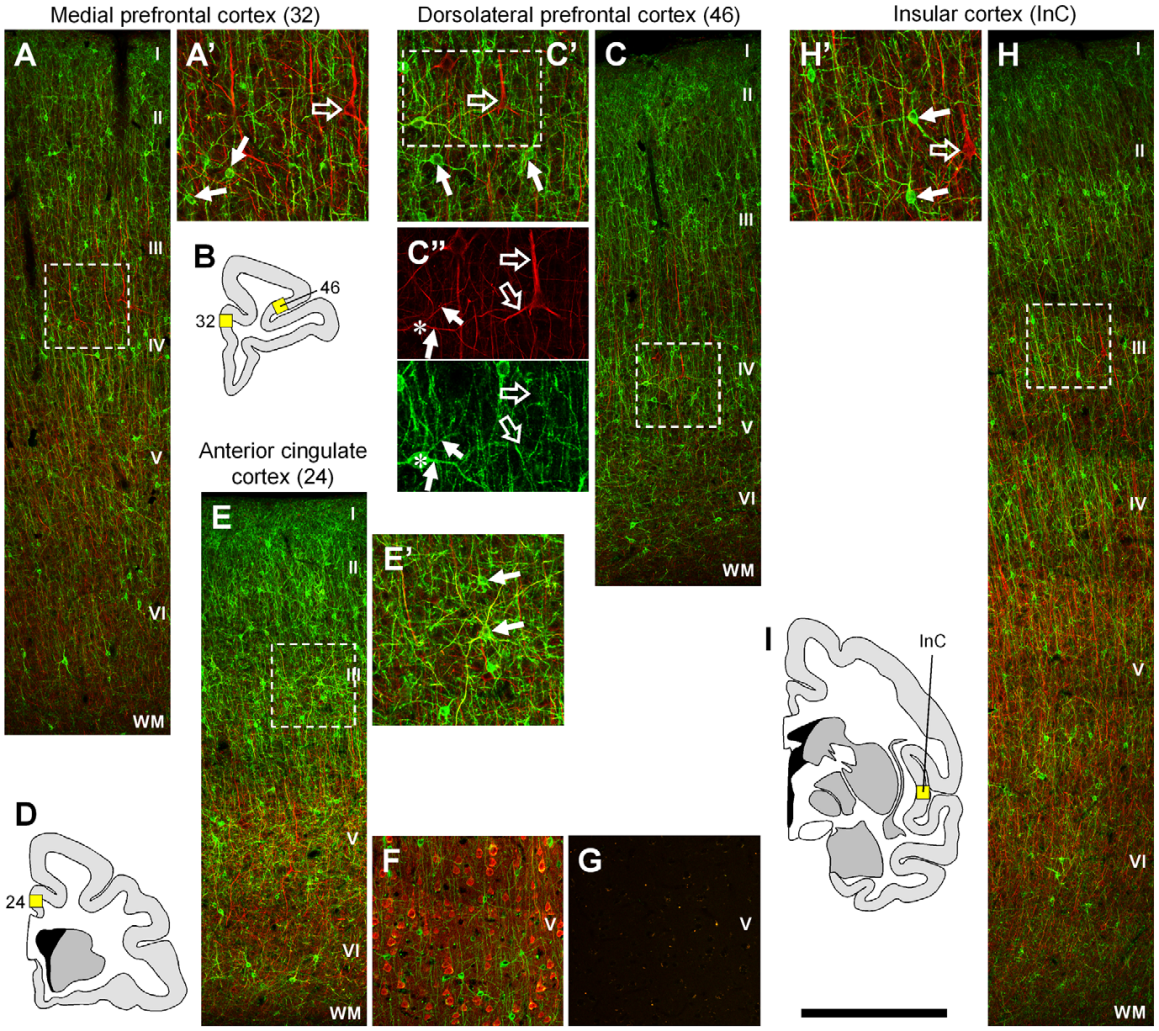
ErbB4 expression in limbic association cortices. The number of ErbB4 expressing cells (mAb-10, green channel) is similarly high in limbic cortices such as areas 32 (A), 46 (C), 24 (E) and insular cortex (H). ErbB4-immunoreactivity is restricted to neuropil in layer I, whereas the number of ErbB4-positive neuronal somata is high in layers II–IV, intermediate in layer V, and low in layer VI. The red channel shows neurofilament H immunofluorescence in panels (A, C, E, H) or neurogranin in (F). Note that immunoreactivity for ErbB4 (arrows) is mostly absent from neurofilament H-positive cells (open arrows), consistent with ErbB4 expression in interneurons. Exceptions are shown in (C”, E'), where dendrites of large ErbB4 positive cells are labeled by neurofilament H. However, the shape of these cells is multipolar and not pyramidal-like and no overlay is detectable using neurogranin as marker for pyramidal cells in area 24 (F). Please note that ErbB4 in (F) was visualized with mouse mAb-77 instead of mAb-10 (A, C, E, H) and a different experimental protocol was used, thus resulting in somewhat weaker immunofluorescence and thinner dendrites. Immunofluorescent signal was entirely absent when primary antibodies were omitted (G); the example is from area 24. The location of the microscope images within the monkey brain is indicated by yellow rectangles in (B, D, I). Images were obtained with a confocal microscope. Scale bar = 400 µm (A, C, E–H), 175 µm (A', C', E', H'), 125 µm (C”).

The entire procedure for immunofluorescent labeling, imaging, image assembly and post-processing was standardized to allow the comparison of expression levels across different sections and different areas throughout the brain. The imaging sensitivity in Figures 1, 2, 3 was chosen to display entire sections; therefore, some brain areas such as the mesencephalic nuclei (Figure 3) show little signal and only in areas of highest epifluorescence the signal was saturated. Please note that the imaging sensitivity was considerably increased in later figures to present ErbB4 immunoreactivity that is still clearly above background levels in the same mesencephalic nuclei at higher magnification using confocal microscopy. Again, in order to allow qualitative comparisons with respect to signal intensity between brain areas within the corticolimbic and the sensory-motor systems, imaging parameters were held constant during imaging of cortical profiles and the basal ganglia, e.g. in Figure 4 (panels A, C, D, F).

Please note that imaging in the dorsal part of the section presented in Figure 2A was not possible in the TH-channel (red) because of a large staining artifact; the green channel was not affected by this event. In order to provide an overview image with realistic color distribution, TH staining in the dorsal cortex was replaced with generic pixel values in the red channel that are representative for the pixel intensity as determined from parallel sections.

### Qualitative analysis of immunofluorescence intensity

Cortical and subcortical areas were identified by analyzing their shape and their location with respect to neighboring structures on series of sections immunolabeled for either tyrosine hydroxylase (TH), GAD67, parvalbumin (PV), neurogranin, or neurofilament H (SMI-32), and in a few cases Nissl-stained sections, and comparison with a rhesus monkey brain atlas [34]. A list of operational definitions of major areas is provided in Table S1. In order to facilitate the identification of cortical areas and subcortical structures, brain sections were selected in order to closely reflect the anterior-posterior levels presented in the brain atlas. A minimum of two sections per area, and in most cases three to five sections per area, were analyzed in both monkeys using identical experimental protocols. In addition both ErbB4 monoclonal antibodies were used on sections from both monkeys. The location of cortical layers was primarily determined using parvalbumin and neurofilament H staining; however, no attempt was made to locate their exact boundaries or to subdivide single layers, as in the case of the highly-structured layer IV of the primary visual cortex (V1).

In the description of signal intensities in different brain areas and cortical layers, arbitrarily set thresholds were used to distinguish *high*, *medium*, and *low* levels of immunoreactivity; occasionally we classified intensity in some regions as *low to medium* or *medium to high* when we found greater than usual variability between sections. The variability of inter-area differences on single sections and between sections that were labeled in a single experiment was smaller than the variability between sections that were immunolabeled in different experiments. Signal intensities were determined by outlining the region of interest and measurement of mean pixel intensity and standard deviation (SD) throughout the entire area under investigation (see Figure S1) whenever its boundaries were detectable using simple morphological criteria (e.g. cortical sulci in the case of the insular cortex) or characteristic immunoreactivity (e.g. tyrosine hydroxylase for the dorsal striatum or parvalbumin for the reticular thalamus). However, since we did not attempt to determine the exact boundaries of all cortical areas of interest, in some cases we instead evaluated fluorescence intensity in 500 µm-wide radial profiles through all cortical layers near the presumed center of the area, in order to stay within its boundaries. Similarly, if the exact boundaries of subcortical structures could not be clearly determined, regions of interest were drawn within the unambiguously identified core structures. Qualitative differences in signal intensities between cortical layers were determined by smoothing the image profile of the cortical area with a Gaussian filter (Passes: 1; Strength: 30) and analysis of a histogram along a radial profile (see Figure S2). Please note that all images presented in the figures were obtained from the same rhesus monkey because of better tissue quality, i.e. consistent absence of both cryoartifacts and sectioning artifacts. However, area-specific mean intensity values were calculated using data from both monkeys, and the sections presented in Figures 1, 2, 3 were selected in order to reflect the entirety of data on mean intensities and inter-area differences closely.

We noticed that high levels of autofluorescence (probably caused by intracellular accumulations of lipofuscin) give the false impression of immunoreactive signal in some areas, e.g. in the lateral geniculate nucleus (Figure 3A); where appropriate, we commented on this signal in the figure legends. Technically, lipofuscin-derived autofluorescence can be distinguished from immunofluorescence by imaging in additional channels (e.g. 405 nm or 635 nm) that should be devoid of immunofluorescent signal from either Alexa 488 / Cy2 or Cy3 fluorophores, which were exclusively used in our experiments. We present examples of this procedure in the external globus pallidus (Figure 2D) and in the lateral geniculate nucleus (Figure 3D) where the far red channel at 635 nm was used to identify strongly autofluorescent somata.

## Results

### ErbB4 immunoreactivity is generally high in the cortex but differs between subcortical structures

We started our analysis of ErbB4 protein expression in the primate brain by collecting and assembling low-magnification epifluorescence images of entire coronal sections in order to get an overview of the general pattern of ErbB4 expression throughout major areas of the monkey cerebrum. The sections were co-immunolabeled for tyrosine hydroxylase (TH) to facilitate identification of the nucleus accumbens, dorsal striatum, and mesencephalic nuclei. In order to allow qualitative comparisons between different sections and brain areas, Figures 1, 2, 3 show sections that were immunolabeled together using the mouse mAb-77 ErbB4 antibody, and data collected using identical microscope settings and standardized post-acquisition image processing (see Methods).

Figure 1A presents ErbB4 expression in a section through the frontal lobe, including the rostral striatum and the nucleus accumbens. ErbB4 immunoreactivity was evident in all cortical areas, with higher overall levels in dorsal and lateral areas compared to ventral cortex. Images presented at original resolution show that cortical immunolabeling occurred in small somata that were scattered throughout the cortex, and in the surrounding neuropil (Figure 1B), consistent with our recent description of selective ErbB4 expression in a subset of interneurons in the prefrontal cortex of rhesus monkeys [12]. Immunofluorescence was largely absent from white matter at low magnification. We detected medium to high levels of ErbB4 in the TH-immunoreactive basal ganglia, including caudate nucleus, putamen, nucleus accumbens, and low to medium levels in the claustrum where TH-innervation was largely absent. In addition, patches of high protein expression occurred frequently in the ventral forebrain (Figure 1C); similar expression patterns in the ventral forebrain have been reported previously for ErbB4 mRNA in rodents [35], [36]. However, in contrast to the cortex, labeling was mostly diffuse in the striatum and ventral forebrain, indicative of expression on neuropil rather than neuronal somata. Thus, additional studies are needed in order to resolve to what extent ErbB4 transcripts and protein may be targeted to somata versus neuropil in these rostral subcortical areas.

Figure 2A shows ErbB4 expression in a section through the rostral thalamus, hypothalamus and amygdala. Again, the striatum can be identified by TH immunofluorescence. The gradient of higher expression levels in dorsal cortex compared to ventral cortex, described in Figure 1, was maintained at this rostrocaudal level, and ErbB4 immunofluorescence was largely absent from cortical white matter and fiber bundles such as the optic tract. We detected medium ErbB4 levels in both the external and internal subdivisions of the globus pallidus in addition to medium to high expression in the striatum, arguing for a potentially important functional role of ErbB4 in subcortical motor signal processing. Medium immunoreactivity was also evident in the reticular thalamic nucleus (RTN). This finding is consistent with mRNA expression in the RTN of rodents as has been shown repeatedly by *in situ* hybridization [7], [35], [37], [38]. Images at original resolution reveal ErbB4 expression in a high number of small somata in cortical layers 2–3 but not in layer 1 (Figure 2B), and in the subcortical claustrum (Figure 2C). Expression in the putamen was restricted to few small somata, and in addition the surrounding neuropil shows medium fluorescence intensity (Figure 2C). This pattern suggests that intraareal ErbB4 expression in the striatum is restricted to interneurons and is absent from principal cells, i.e. medium spiny neurons, similar to the cerebral cortex. Both the intercalated nuclei of the amygdala and the ventral hypothalamus displayed high ErbB4-immunofluorescence, consistent with previous reports in rodents [7], [35], [39]. Results from previous studies suggest that the ventral hypothalamic signal may derive from astrocytes rather than neurons [40]–[42]. We routinely executed imaging controls in order to distinguish immunofluorescent signal from non-specific autofluorescence (e.g. accumulations of lipofuscin in cellular somata). Figure 2D presents an example from the external globus pallidus (see Methods for details).

In Figure 3A we present ErbB4 expression in a section through the hippocampal formation, posterior thalamus, and mesencephalon. The overview image shows that the overall level of ErbB4 immunofluorescence was clearly lower in the hippocampal formation compared to dorsal neocortical areas. However, higher magnification reveals that expression was high in multipolar cells scattered throughout the hippocampus. It is also evident that many cells in the subgranular zone located at the border between the granule cell layer and the hilus were ErbB4-positive (Figure 3B). With exception of the RTN, ErbB4 protein expression was low in the thalamus, intermediate in the central gray nucleus, and again low in the substantia nigra pars compacta (Figure 3C). Figure 3D presents another example for detection of autofluorescence from the lateral geniculate nucleus.

### ErbB4 expression is high in areas of the “corticolimbic system”

Next, we investigated ErbB4 expression in more detail by using confocal microscopy to increase magnification and resolution, and by using different neuronal markers in double-immunofluorescence to identify neuronal subtypes. We present our findings arranged in functional systems, and we start with areas of the “corticolimbic system” [43]–[45] that are important for the proper execution of different types of memory (prefrontal cortex, hippocampus), attention and goal-directed behavior (prefrontal and anterior cingulate cortex), and emotion and reward (amygdala, nucleus accumbens). These different areas have also been implicated in the etiology of schizophrenia [46]–[48].

We began by analyzing the prefrontal cortex using double-immunofluorescence for ErbB4 and a principal neuron marker (Figure 4). ErbB4 was detected with a rabbit monoclonal antibody (mAb-10) directed against an intracellular epitope [11] and principal neurons were labeled with an antibody raised against neurofilament H [32]. We found high expression levels of ErbB4 in somata and dendrites of many multipolar neurons scattered throughout layers 2–6 and a few immunoreactive interstitial neurons in white matter. This pattern was largely identical in medial and dorsolateral prefrontal cortex (Figure 4A, C), anterior cingulate cortex (Figure 4E), and insular cortex (Figure 4H). We verified with an additional marker that ErbB4 immunoreactivity was consistently absent from pyramidal neurons (Figure 4F). Figure 4G shows absence of immunofluorescence in control experiments.

**Figure 5:**
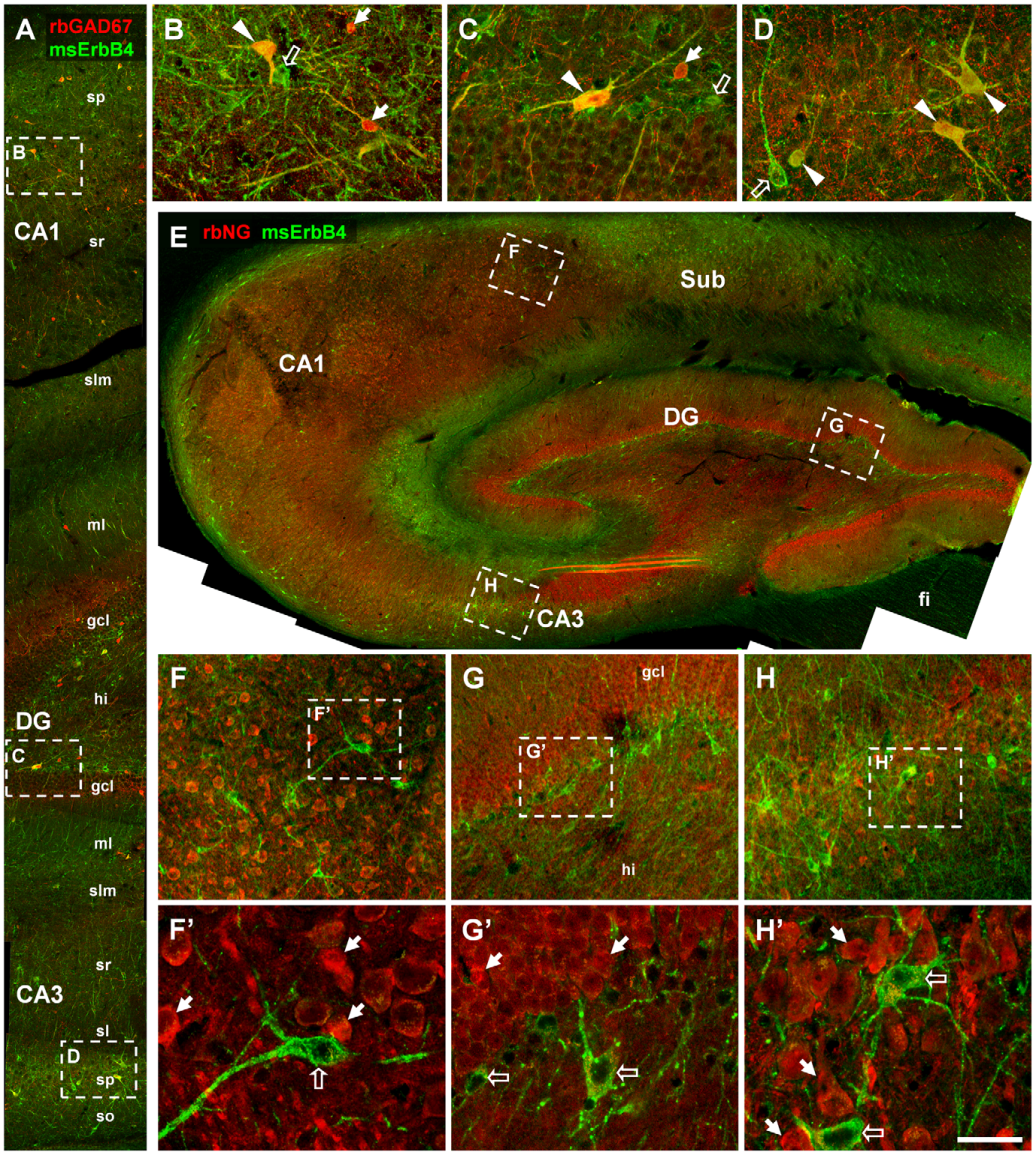
ErbB4 expression in the hippocampal formation. (A–D) Double-immunofluorescence reveals that many ErbB4-positive neurons (mAb-77, green channel) coexpress GAD67 (red channel; arrowheads in B–D). Moreover, the multipolar shape of neurons implicates interneurons rather than pyramidal cells, even if some ErbB4-positive cells do not express GAD67 (open arrows). Note that not all GAD67-positive neurons express ErbB4 in the soma (arrows in B–C). The overview of the hippocampal formation (E) using a rabbit neurogranin antiserum (rbNG) to identify principal neurons, shows low concentration of ErbB4 in principal cell layers of cornu ammonis and DG, but high expression in molecular layers and a high number of ErbB4-positive somata in the basal stratum oriens of CA1 and throughout the Sub. (F–H) Consistently, we find a lack of coexpression of ErbB4 (open arrows in F'–H') and the principal cell marker neurogranin (arrows F'–H') in CA1-3 and in DG. Images were obtained with a confocal microscope. Scale bar = 200 µm (A), 50 µm (B–D), 470 µm (E), 100 µm (F–H), 32 µm (F'–H'). Abbreviations: Cornu ammonis 1/3 (CA1/3), dentate gyrus (DG), fimbria (fi), granule cell layer (gcl), hilus (hi), molecular layer (ml), stratum lacunosum molecular (slm), stratum oriens (so), stratum pyramidale (sp), stratum radiatum (sr), subiculum (Sub).

Next, we analyzed ErbB4 expression in the hippocampal formation using combinations of mouse mAb-77 with rabbit antibodies against either GAD67, in order to identify GABAergic interneurons, or neurogranin, which labels glutamatergic principal cells. A profile through the major subregions of the hippocampus (Figure 5A) shows that ErbB4 is expressed in many GAD67-positive neurons in the CA1 (Figure 5B), dentate gyrus (Figure 5C), and CA3 (Figure 5D). ErbB4 is also expressed in many neurons in the deep stratum oriens close to the alveus and in the subgranular zone of the dentate gyrus (Figure 5E). In addition, ErbB4-positive cells are detected at lower density throughout all hippocampal layers (Figure 5F–H). Please note the lack of ErbB4 and neurogranin coexpression (Figure 5E–H), indicating that receptor expression is restricted to interneurons, consistent with our findings in the mouse hippocampus [10].

### ErbB4 expression in areas of sensory and motor systems

**Figure 6:**
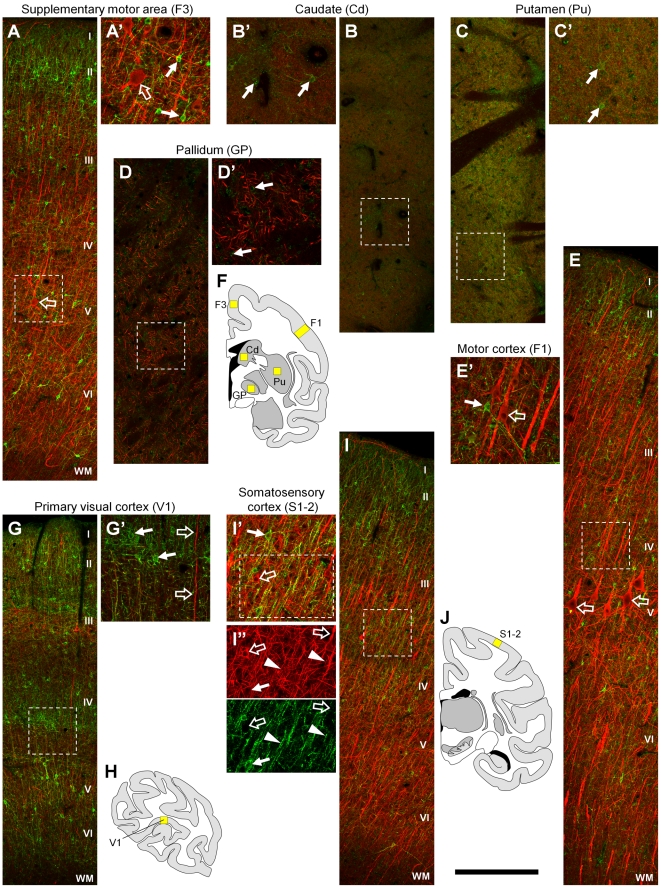
ErbB4 expression in sensory-motor areas. In both the supplementary (A) and the primary motor cortex (E), ErbB4-positive cells are concentrated in layer II. ErbB4-immunoreactivity (mAb-10, green channel) is restricted to small multipolar cells (arrows) but is absent from the large Betz cells that are neurofilament H-positive layer V pyramidal neurons (red channel, open arrows in A, A', E, E'). ErbB4 expression in the basal ganglia is generally more diffuse and mostly lower compared to the cortex. Within the different nuclei, expression is intermediate in the caudate (B), highest in the putamen (C), and lowest in pallidum (D). Higher magnification shows that the expression on individual cells is low (B'–D'). In the primary visual cortex (G, G'), ErbB4 expression is higher compared to other sensory cortical areas and high numbers of immunoreactive somata are evident in layers II and upper layer III, as well as in layer IV-Cα. Intensely-labeled larger somata are spread throughout layer V and, at lower density, layer VI. In the somatosensory cortex (I), ErbB4 is mostly expressed in apical layers I–III. In either sensory cortex (G', I', I”), higher magnification shows that ErbB4 expression is restricted to multipolar cells (arrows) but is absent from pyramidal cell somata and thick dendrites (open arrows). Some ErbB4 expression occurs on small diameter neurofilament H-positive neurites (arrowheads). The location of the microscope images within the monkey brain is indicated by yellow rectangles in (F, H, J). The red channel shows neurofilament H expression in all panels. Images were obtained with a confocal microscope and imaging parameters were held constant between panels A–E, G, I to allow the comparison of signal intensities between different areas. Scale bar = 400 µm (A–E, G, I), 175 µm (A'–E', G', I'), 125 µm (I”).

We next investigated ErbB4 immunoreactivity in primary sensory cortical areas (visual V1, somatosensory S1) and major areas of the motor system (primary motor cortex F1, supplemental motor cortex F3, basal ganglia) using mAb-10 and neurofilament H antibodies in double-immunofluorescence experiments. Our results show generally lower levels of ErbB4 expression in both motor (Figure 6A, E) and sensory (Figure 6G, I) cortices, as compared to frontal cortical areas (cf. Figure 4). Expression was usually medium to high in apical layers but much weaker in basal layers, with the notable exception of area V1 that showed two broad bands of high expression in apical and in basal layers, and only sparse expression in between (Figure 6G). Nuclei of the basal ganglia, related to motor function, displayed different levels of ErbB4 immunoreactivity. The strongest immunofluorescence was detected in putamen (Figure 6C), followed by the caudate nucleus (Figure 6B), and less signal in the pallidum (Figure 6D). Higher magnification shows that all three nuclei display sparsely distributed multipolar cells with relatively weak ErbB4 expression compared to cortical interneurons. Most of the immunofluorescence in caudate and putamen represents diffuse signal consistent with expression on neuropil that might potentially originate from dopaminergic axons. Of note, the signal intensity in the basal ganglia appears to be much lower compared to the overview images presented in Figures 1 and 2. This difference is mainly due to the fact that the low-resolution overview images in Figures 1, 2, 3 integrate signal from somata and background. In addition, these images were taken with a 10x objective using epifluorescence, resulting in signal collection practically across the entire depth of the section. In contrast, the full resolution images in Fig. 6B–D were obtained with a 20x objective on a confocal microscope (collecting signal from a much thinner z-plane) to allow the separation of individual somata from the surrounding background.

### ErbB4 is expressed in different subpopulations of cortical interneurons

**Figure 7:**
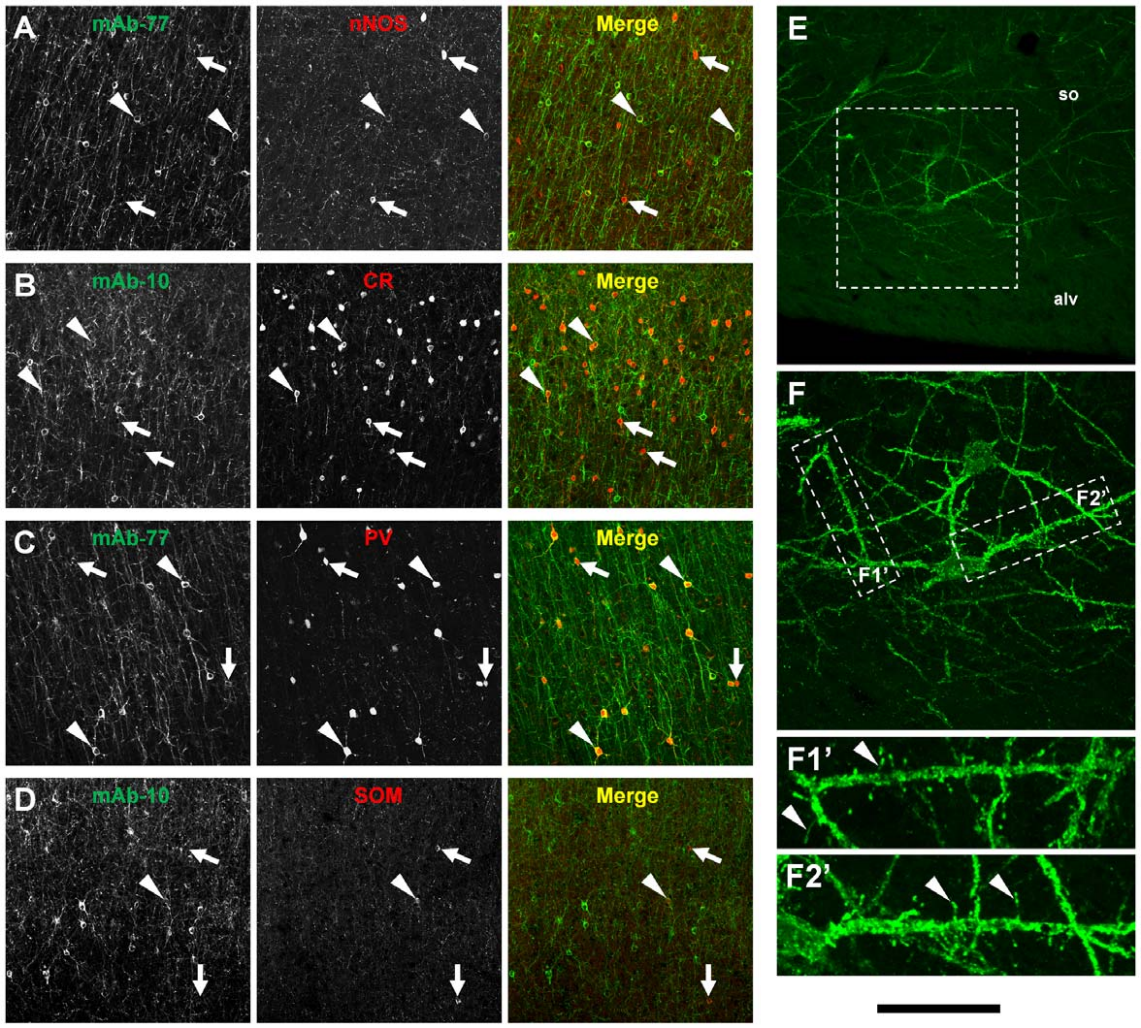
ErbB4 is expressed in different populations of cortical interneurons. Using double-immunofluorescence we detected ErbB4 expression (mAb-10 or mAb-77, green channel) in different subsets of interneurons (red channel) in the insular cortex. (A) Interneurons with low nNOS-immunoreactivity showed coexpression of ErbB4 (arrowheads), whereas high-expressing cells were consistently ErbB4 negative (arrows). (B) Many CR-positive interneurons in layers 2–3 were immunoreactive for ErbB4 (arrowheads). (C) Almost all PV neurons coexpressed ErbB4 throughout all cortical layers. (D) The number of SOM-positive interneurons was low in the insular cortex, and a subset coexpressed ErbB4. (E) Occasionally, we detected ErbB4 in a population of hippocampal spiny interneurons in lower stratum oriens of the CA3 region. (F, F1-2′) Higher magnification shows ErbB4 immunofluorescence within the somatodendritic compartment including the spines. Scale bar = 200 µm (A–D), 120 µm (E), 60 µm (F), 35 µm (F1-2′). Abbreviations: alveus (alv), calretinin (CR), neuronal nitric oxide synthase (nNOS), parvalbumin (PV), somatostatin (SOM), stratum oriens (so).

Here, we have shown ErbB4 expression in hippocampal interneurons (Figure 5) and absence of ErbB4 immunofluorescence from pyramidal cells in many cortical areas, including examples of sensory, motor, and association cortices (Figs 4, 6). Moreover, we have recently identified several different subtypes of ErbB4-positive interneurons in the frontal cortex of monkeys [12]. In order to verify this expression pattern in an additional area of the primate cortex we went on to analyze cellular ErbB4 expression in the insula (Figure 7A–D). Using double-immunofluorescence we detected ErbB4 protein in all four different subtypes of GABAergic interneurons investigated, namely those expressing neuronal nitric oxide synthase, calretinin, parvalbumin, and somatostatin. The distribution of ErbB4 in the insula is thus similar to the prefrontal cortex of monkeys, and is also consistent with expression in hippocampal interneurons, suggesting that this pattern might be representative for the cerebral cortex of monkeys.

In addition, we detected ErbB4-immunoreactivity in a small population of spiny interneurons in the stratum oriens of hippocampal CA3 (Figure 7E, F). The multipolar shape, the tangential orientation, and the location in the stratum oriens of CA3 are indicative of interneurons and are consistent with a previous description in rodents [49]. We found these cells on several sections from either of the two monkeys; however, their number was always low and we did not verify whether they might be GABAergic, cholinergic, or glutamatergic. Of note, these cells in stratum oriens should not be confused with the well known glutamatergic spiny interneurons in stratum lucidum of CA3 [50] or with the population of GABAergic spiny interneurons that have been described in the hippocampal hilus [51]. In any case, the identity and function of these neurons in stratum oriens and the role of ErbB4 in their spines are unclear and warrant further investigation.

### ErbB4 expression in subcortical limbic structures: Amygdala and nucleus accumbens

**Figure 8:**
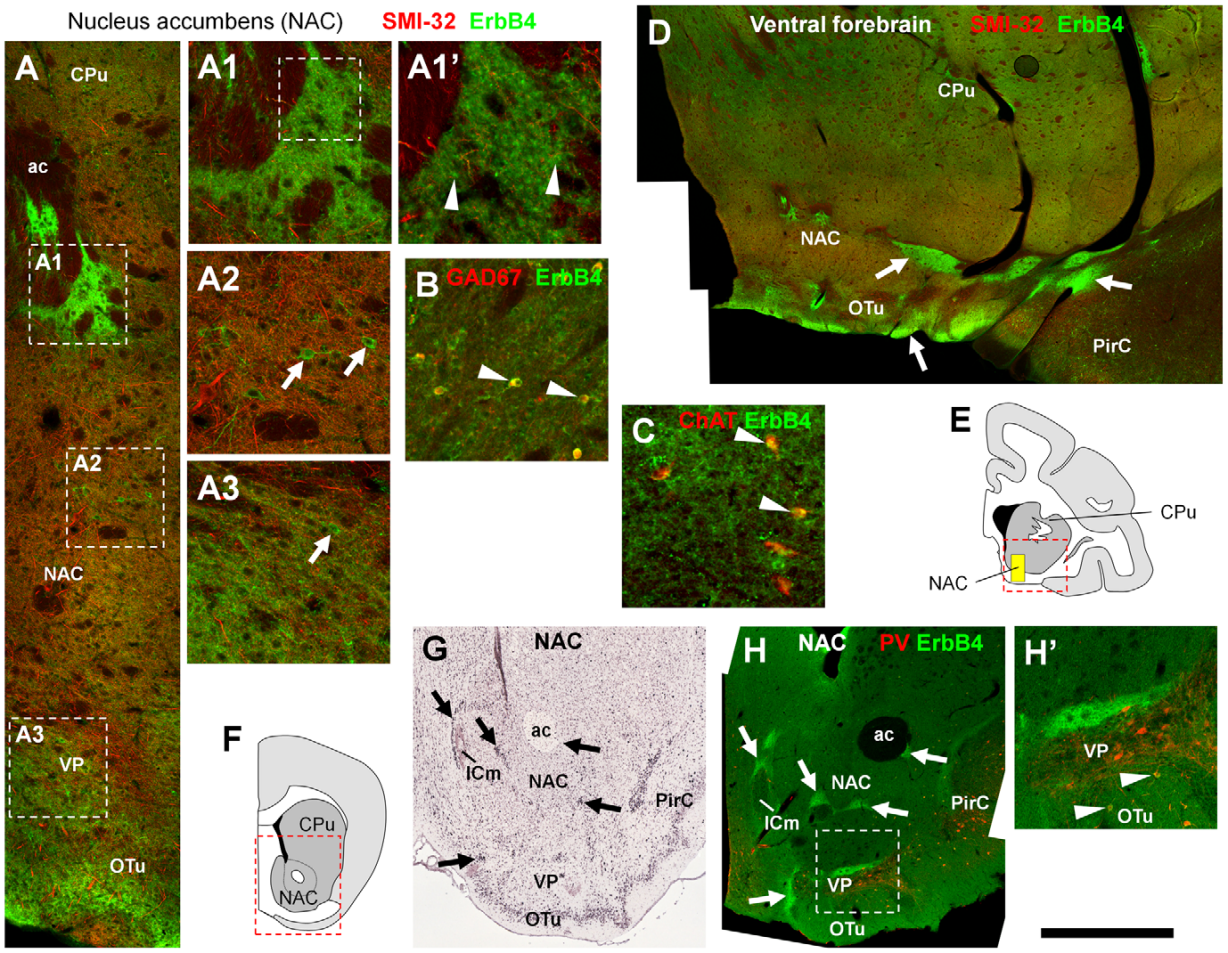
Consistent pattern of high-level ErbB4 expression in the ventral striatum of monkeys and mice. Overviews on the ventral striatum and basal forebrain in the monkey (D) and the mouse (H) show similar patterns of patches of high ErbB4 (mAb-10, green channel) immunoreactivity (arrows). Higher magnification (A, A1, A1', H') reveals that in these patches ErbB4 is located on neuropil and glomeruli-like structures or, possibly, on small somata (arrowheads in A1'). In contrast, larger ErbB4-expressing somata reside in the NAC and VP outside of the patches (arrows in A2, A3). Double-immunofluorescence labeling in the NAC shows that most ErbB4-positive somata coexpress GAD67 (B), whereas a subset coexpresses ChAT (C); arrowheads label coexpressing neurons. In the mouse, the pattern of ErbB4 protein expression (H) is largely consistent with mRNA expression (G) as obtained from the Allen Brain Atlas (Seattle, WA: Allen Institute for Brain Science. © 2004–2011. Available from: http://www.brain-map.org). The red channel shows immunofluorescence for neurofilament H (SMI-32) in (A, A1–A3, A1', D), GAD67 (B), or ChAT (C) in the monkey, and parvalbumin expression in the mouse (H, H'). Note ErbB4-parvalbumin coexpressing cells in VP (H', arrowheads). The locations of the overview images within the monkey (D) and mouse (H) brain are indicated by red rectangles in the respective drawings (E, F). The location of the higher magnification profile (A) is indicated by the yellow box in (E). Images were obtained with a confocal microscope except for (B, C) that were taken by epifluorescence microscopy. Scale bar = 2.4 mm (D), 1.2 mm (H), 540 µm (A), 300 µm (H'), 150 µm (A1–A3, B, C), 60 µm (A1'). Abbreviations: anterior commissure (ac), choline acetyltransferase (ChAT), caudate-putamen (CPu), major island of Calleja (ICm), nucleus accumbens (NAC), olfactory tubercle (OTu), piriform cortex (PirC), ventral pallidum (VP).

Next, we investigated subcortical areas of the limbic system that were previously shown to express high levels of ErbB4 mRNA in rodents [7], [36]–[38]. As shown in Figure 8, ErbB4 immunoreactivity occurs in multipolar neurons in the nucleus accumbens and ventral pallidum (Figure 8A2, A3); while most of these neurons are GABAergic (Figure 8B), we found that a subset is cholinergic (Figure 8C). In addition, we detected very high ErbB4 immunoreactivity in patches throughout the nucleus accumbens and additional structures of the rostral ventral forebrain, both in monkeys (Figure 8A, D) and in mice (Figure 8H). The location suggests that some of these patches could represent islands of Calleja, areas of the ventral pallidum and the rostral migratory stream, that are known to express high levels of ErbB4 in rodents [22], [52]. The general pattern of high ErbB4 immunoreactivity in patches is very similar for mRNA, as evident from an image showing *in situ* hybridization in mouse nucleus accumbens (Figure 8G) obtained from the Allen Brain Atlas [25]. These observations warrant further investigation in order to unequivocally classify ErbB4-expressing cells in these structures and to analyze the subcellular targeting of the receptor within somata and neuropil.

**Figure 9:**
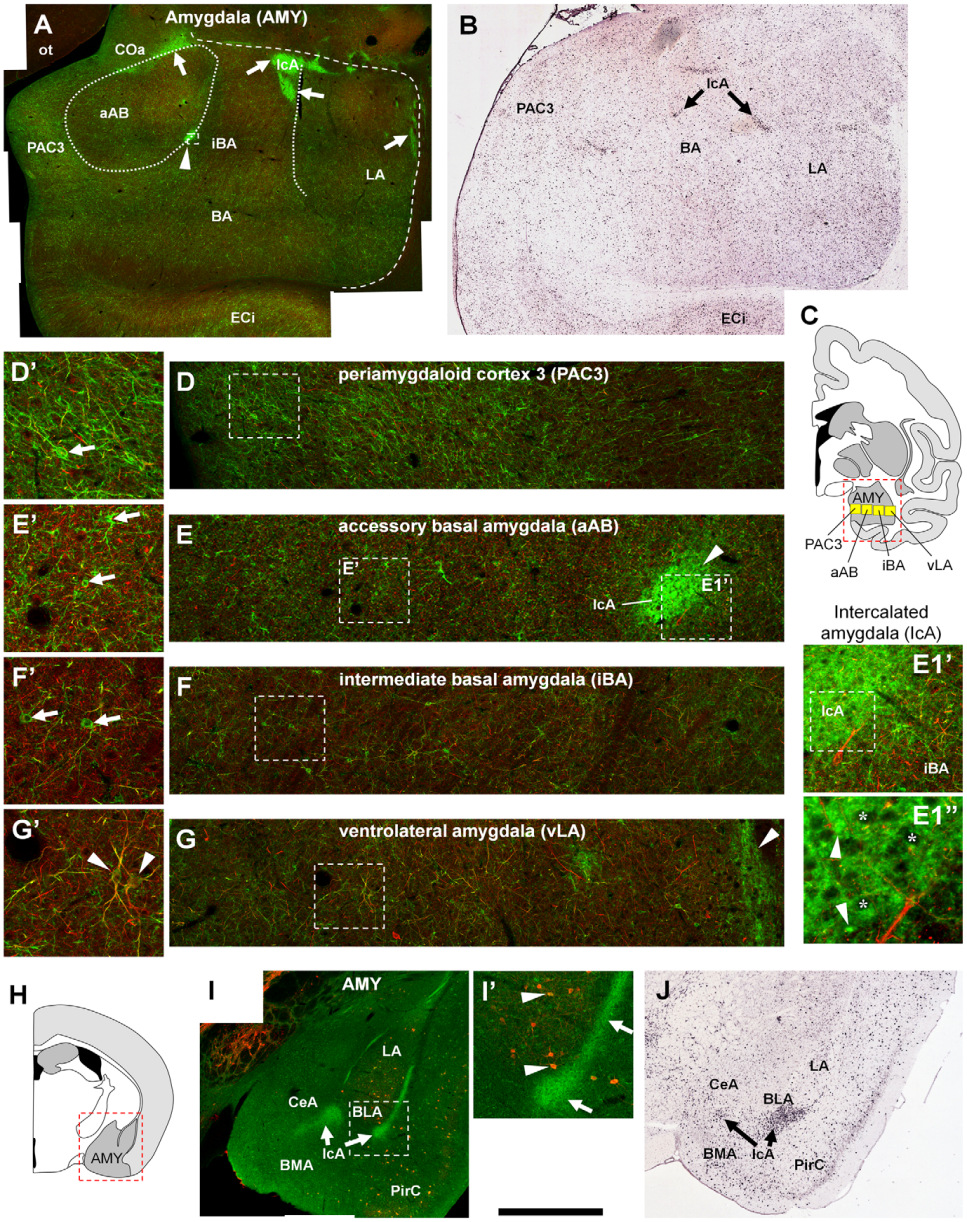
ErbB4 protein expression in the amygdala of monkeys and mice. Overview images show that both ErbB4 protein (A) and mRNA (B) are highly expressed in patches in the amygdala that correspond to the intercalated nuclei. Higher magnification reveals a higher number of ErbB4-positive neurons (mAb-10, green channel) in the cortical amygdala PAC3 (D) compared to the subcortical nuclei (E–G); examples of neurons are indicated by arrows in (D'–G'). Further magnification shows a pattern of dense ErbB4 immunoreactivity surrounding immunonegative cell somata in the intercalated nuclei (somata labeled with asterisks in E1”) and labeling of glomeruli-like structures or, possibly, small somata (arrowheads in E1”). The red channel shows neurofilament H-immunofluorescence in the monkey (A, D–G) and parvalbumin expression in the mouse (I). Please note ErbB4-parvalbumin coexpressing cells in BLA (arrowheads in I'). The location of the overview images within the monkey (A, B) and mouse (I, J) brain is indicated by red rectangles in the drawings (C, H); the position of the profiles through the monkey amygdaloid nuclei (D–G) is indicated by yellow boxes in (C). Images were obtained with a confocal microscope. Images on expression of ErbB4 mRNA in the monkey (B) and the mouse (J) amygdala were obtained from the Allen Brain Atlas (Seattle, WA: Allen Institute for Brain Science. © 2004–2011. Available from: http://www.brain-map.org). Scale bar = 2.4 mm (A, B), 1.2 mm (I), 400 µm (I'), 150 µm (D'–G', E1'), 60 µm (E1”). Abbreviations: accessory basal amygdala (aAB), basal amygdala (BA), basolateral amygdala (BLA), basomedial amygdala (BMA), central amygdala (CeA), anterior cortical amygdala (COa), internal entorhinal cortex (ECi), intermediate basal amygdala (iBA), intercalated amygdala (IcA), lateral amygdala (LA), optic tract (ot), periamygdaloid cortex 3 (PAC3), piriform cortex (PirC), ventrolateral amygdala (vLA).

With respect to the amygdala, we found patches of high ErbB4 immunoreactivity in the monkey (Figure 9A), and detection of mRNA (obtained from the Allen Brain Atlas) reveals a similar pattern (Figure 9B). We suggest that these patches correspond to the inhibitory intercalated nuclei of the amygdala. High magnification revealed structures indicative of ErbB4 expression in large glomeruli-like structures or small somata that are surrounded by intense and diffuse immunofluorescence (Figure 9E1', E1”). This result is consistent with previous reports in rodents [7], [35], [37], and is again supported by immunofluorescent labeling (Figure 9I) and the Allen Brain Atlas (Figure 9J). Besides the intercalated nuclei, the cellular expression pattern of ErbB4 was similar to other cortical areas in the periamygdaloid cortex 3 (Figure 9D). In subcortical nuclei of the amygdala, however, immunoreactive cells were sparsely distributed and overall expression levels were lower (Figure 9E–G). Occasionally, we found co-immunofluorescence of ErbB4 and neurofilament H (Figure 9G'), raising the possibility that a small subset of cells other than inhibitory interneurons might express ErbB4 in subcortical nuclei of the amygdala.

### ErbB4 expression in diencephalic and mesencephalic nuclei

**Figure 10:**
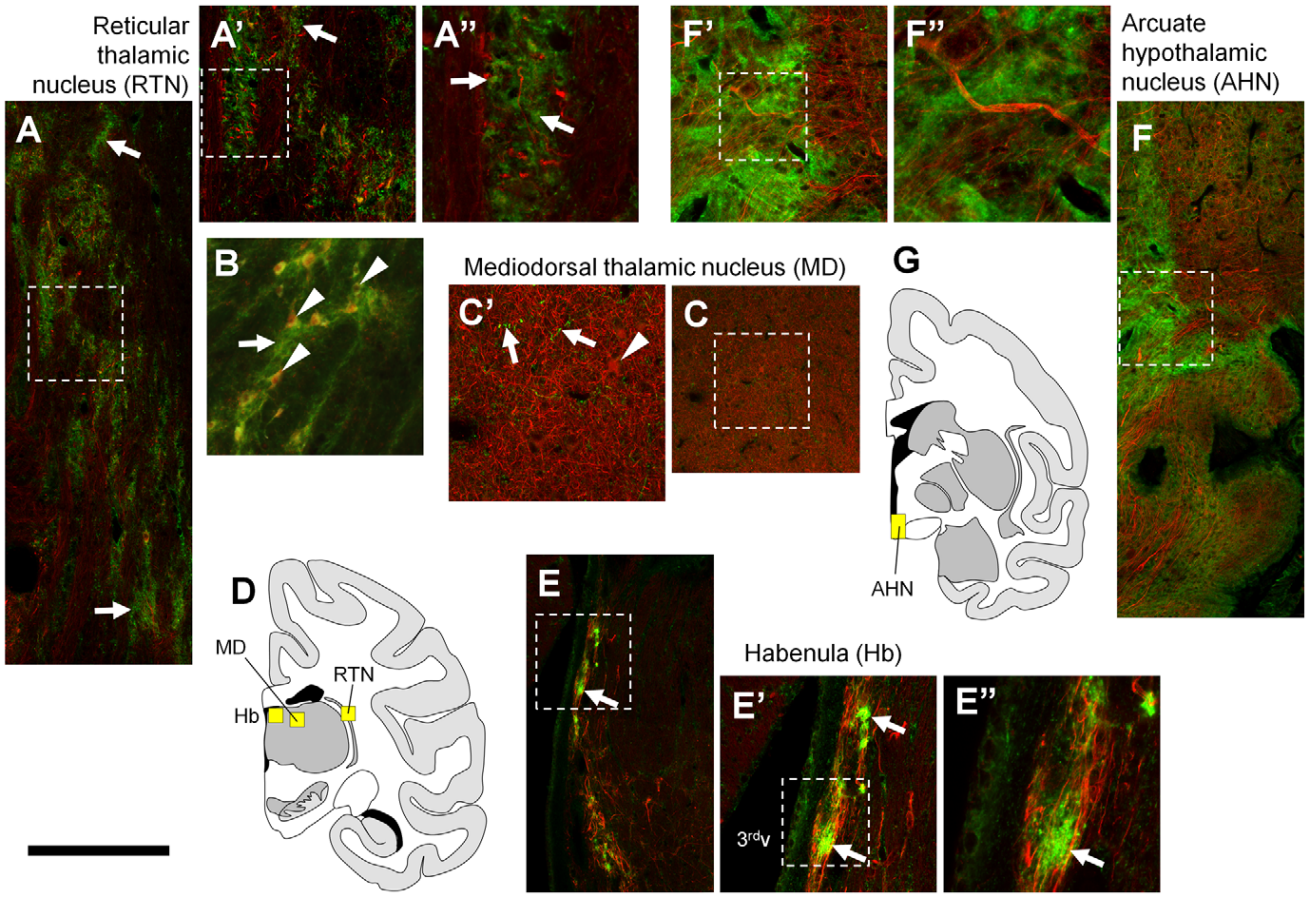
ErbB4 protein expression in diencephalic nuclei. High ErbB4 (mAb-10, green channel) expression (arrows) occurs in the thalamic RTN (A) and habenula (E). Higher magnification shows ErbB4 expression on glomeruli-like structures (arrows in A', A”) and on parvalbumin-positive neuronal somata (arrowheads in B) in the RTN that are surrounded by neuropil (arrows in A, B). We detected patches of high ErbB4 expression but could not identify cellular structures in the habenula even at high magnification (arrows in E', E”). In contrast, ErbB4 is almost absent from the MD thalamus (C) except for immunoreactivity on a few neurites (arrows in C') but not on somata (arrowhead in C'). We additionally found high expression in neuropil of the arcuate hypothalamic nucleus of the monkey (F–F”). The red channel shows neurofilament H-immunofluorescence in panels (A, C, E, F) and parvalbumin in (B). The location of the microscope images within the monkey brain is indicated by yellow boxes in (D, G). Images were obtained with a confocal microscope, except for (B) that was taken by epifluorescence microscopy. Scale bar = 400 µm (A, C, E, F), 175 µm (A', C', E', F'), 150 µm (B), 70 µm (A”, E”, F”).

We next investigated ErbB4 protein expression in the diencephalon and mesencephalon since high levels of mRNA have repeatedly been reported in the rodent reticular thalamic nucleus, habenula, ventral hypothalamus, and in the dopaminergic substantia nigra and ventral tegmental area [7], [9], [35], [38], [53], [54]. Consistent with these studies, we detected medium levels of ErbB4 protein on both neurites and neuronal somata throughout the reticular thalamic nucleus (Figure 10A); virtually all somata were immunoreactive for parvalbumin (Figure 10B). Very high immunoreactivity occurred in small spots in the habenula (Figure 10E); however, we could not identify any cellular structures even at 100x magnification using confocal microscopy. We also investigated the mediodorsal thalamic nucleus (Figure 10C) because it projects specifically to the prefrontal cortex and is thus considered part of the “limbocortical” network of areas that generally showed high levels of ErbB4 expression (cf. Figures 4, 5, 8, 9), but found ErbB4-immunoreactivity to be largely absent. High ErbB4 expression occurred in the arcuate hypothalamic nucleus (Figure 10F); high magnification revealed ErbB4 expression in large glomeruli-like structures or small somata in this area that are surrounded by intense and diffuse immunofluorescence. Expression of ErbB4 has been reported in hypothalamic astrocytes that are involved in the neuroendocrine regulation of sexual maturation and reproductive capacity of rodents [40], [41], and *in vitro* analysis of human fetal astrocytes has indicated that hypothalamic but not cortical astrocytes express ErbB4 [42]. Thus, these studies suggest that our finding of high immunoreactivity in the hypothalamus of rhesus monkeys likely results from expression in astrocytes rather than neurons. Taken together, contrary to the expression pattern in the cortex, the patches of very high immunoreactivity in the rostral ventral forebrain, the intercalated nuclei of the amygdala, selected thalamic nuclei, and the ventral hypothalamus displayed diffuse labeling, and few clearly defined structures could be identified even at high magnification using immunofluorescence. Further studies with additional markers and the use of electron microscopy are needed to identify the exact source of ErbB4 immunoreactivity in these areas.

**Figure 11:**
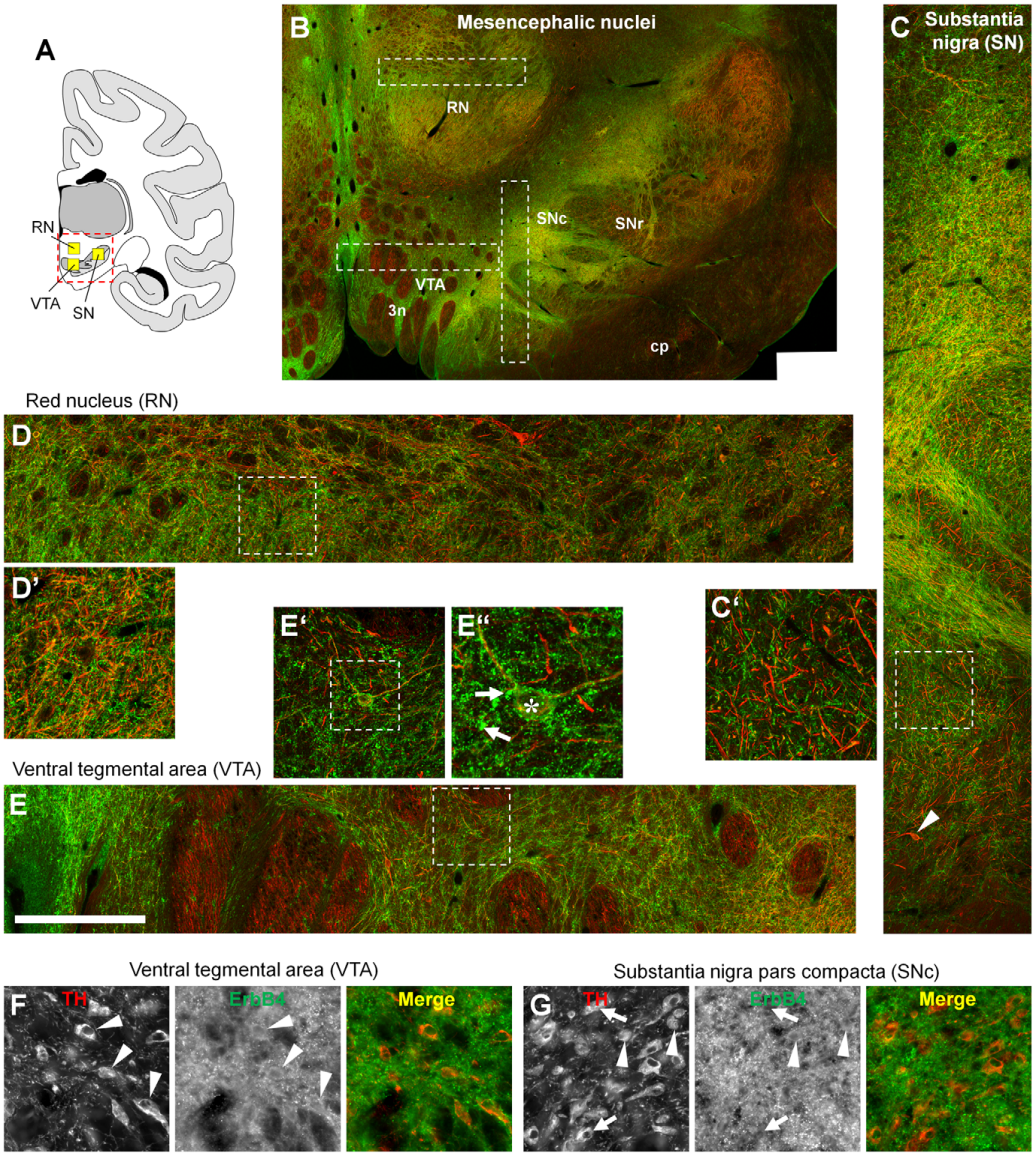
ErbB4 protein expression in mesencephalic nuclei. The overview on the mesencephalon (B) shows dense ErbB4 expression (mAb-10, green channel) in the dopaminergic nuclei SNc and VTA that is clearly above background levels, whereas expression in the red nucleus is weaker. Higher magnification shows abundant immunoreactivity on neuropil (C–E, C'–E'). At 100x magnification, a dense network of ErbB4-positive neuropil surrounding a neuron (asterisk) is evident in the VTA (E”). The red channel shows neurofilament H-immunofluorescence in panels (B–E) and TH-immunofluorescence in (F–G). Co-immunolabeling reveals that subsets of TH-positive dopaminergic somata express ErbB4 in the VTA (F) and SNc (G); coexpressing neurons are indicated by arrowheads (F–G), whereas lack of ErbB4 signal is indicated by arrows (G). The location of the overview image within the monkey brain is indicated by a red rectangle in (A). The location of the profiles through mesencephalic nuclei is indicated by yellow boxes in (A) and by rectangles in (B). Images were obtained with a confocal microscope. Scale bar = 3 mm (B), 400 µm (C–E), 175 µm (C'–E', F–G), 70 µm (E”). Abbreviations: cerebral peduncle (cp), oculomotor nerve (3n), substantia nigra pars reticulata (SNr).

An overview on the mesencephalon shows that ErbB4-immunoreactivity was clearly above background level in the red nucleus (i.e., nucleus ruber), substantia nigra pars compacta (SNc), and ventral tegmental area (VTA) (Figure 11B); however, please note that the absolute level of immunofluorescence is much lower than in the cortex because the confocal detector sensitivity during microscopic image acquisition was strongly increased compared to Figure 3. The overall ErbB4 signal intensity was highest in the SNc, where the highest density of ErbB4 mRNA expressing cells were observed by in situ hybridization [7], [35], [37], [38]. Higher magnification reveals that most of the signal derived from structures reminiscent of neuropil in all three nuclei (Figure 11C–E). However, low-level ErbB4 immunoreactivity was evident on a subset of TH-positive dopaminergic neuronal somata in the VTA and SNc (Figure 11F–G).

## Discussion

Our study represents the first extensive analysis of ErbB4 protein expression throughout major areas of the primate cerebrum. In order to emphasize the potential functional role of ErbB4 receptors, the results on their expression patterns were organized according to anatomical considerations in areas associated with corticolimbic, sensory and motor functions in the cortex, and associated with the subcortical telencephalon, diencephalon and mesencephalon. For this study we used two highly specific and well characterized monoclonal antibodies, targeting either an extracellular or an intracellular epitope of ErbB4, that produce essentially identical patterns of immunoreactivity [10]–[12]. We found a strong correlation between protein expression and previous reports on mRNA expression in most areas, despite the fact that most published data on ErbB4 mRNA were derived from rodents. This finding prompted us to include parallel experiments in monkey and mouse nucleus accumbens and amygdala that yielded very similar patterns of protein expression in both species. These results are consistent with our prior findings in the frontal cortex of mice, rats, monkeys and humans that also showed regional and cellular conservation of ErbB4 expression across species [12], suggesting an evolutionarily conserved pattern of ErbB4 expression in the brain. However, we cannot exclude the possibility that future studies, using other markers or methods (i.e., electron microscopy), may reveal species-dependent differences in cellular expression and subcellular targeting of ErbB4, since this initial assessment is based on experiments using a limited number of monkeys, markers and mostly low-resolution approaches.

### Comparison of ErbB4 expression in rodents and primates

Several studies have investigated ErbB4 mRNA expression in rodents by *in situ* hybridization and, besides dot-patterned expression throughout the entire cerebral cortex, consistently reported on high subcortical expression levels in the ventral forebrain, amygdala, habenula and reticular thalamic nucleus, ventral hypothalamus, and ventral mesencephalon [7], [35], [37], [38]. Similar expression patterns are presented in the Allen Brain Atlas [25], and we have included examples from the amygdala and nucleus accumbens in the results. The expression of ErbB4 in rodent TH-positive mesencephalic neurons was shown by *in situ* hybridization. Moreover, simultaneous decreases of both mRNAs during adulthood and aging [55] or following 6-hydroxydopamine-induced dopaminergic lesion [35] were reported in rats, further arguing for ErbB4 expression in dopaminergic neurons. With respect to ErbB4 protein, several studies have provided detailed analyses of cellular and subcellular expression in rodent interneurons of the neocortex, hippocampus and olfactory bulb [8], [10], [11], [15], [22]–[24], but little is known about expression in subcortical structures except for the ventral mesencephalon (see above) and the ventral hypothalamus [40], [41].

In primates, including humans, much less data are presently available. Several studies investigated ErbB4 protein expression in small cortical tissue samples of humans and primates, and they reported widespread protein expression on both interneurons and principal cells [26]–[28]. However, we have recently demonstrated that the antibodies used in those studies exhibit major unspecific cross-reactivity in monkey and human tissues [12]. Instead, we showed that in the frontal cortex of monkeys and humans ErbB4 is selectively expressed in interneurons and is absent from pyramidal cells, consistent with findings in rodents [8], [10], [11]. Our current study has extended these investigations by including many functionally and cytoarchitecturally heterogeneous cortical regions where the distribution, size and shape of ErbB4-immunoreactive cells is consistent with expression in inhibitory interneurons. Moreover, expression in GABAergic interneurons was directly demonstrated in the hippocampus by double-immunofluorescence with GAD67, and in the insular cortex with calcium binding proteins and the neuropeptide CCK. Thus, our results suggest that the pattern of interneuron-specific expression of ErbB4 is maintained throughout the entire cerebral cortex in primates.

With respect to additional subcortical regions in the human brain, ErbB4 mRNA is expressed in the ventral mesencephalon, and indirect evidence suggests that the ventral hypothalamus expresses high levels of ErbB4 [42]. Our current analysis generally supports these findings which are also consistent with data available from the Allen Brain Atlas [25]. This database provides information obtained by *in situ* hybridization on ErbB4 expression in several species, including rhesus monkeys and humans, but only few regions of the monkey and human brain have been investigated yet.

### ErbB4 expression in different cell types

Generally, our results obtained from various regions of the cerebral cortex are consistent with the selective expression of ErbB4 in interneurons (see above). The primary visual cortex (V1) showed high to medium levels of ErbB4 expression in two broad bands roughly encompassing layers 2–3 and lower layer 4 to layer 6, respectively, and lower expression in between. This pattern is reminiscent of the distribution of parvalbumin (PV)-positive interneurons in V1 [56]. These findings, together with previous reports on cell type-specific cortical expression in both primates [12] and rodents [8], suggest that PV-positive cells probably comprise the largest subpopulation of ErbB4-expressing interneurons in the neocortex. We also detected ErbB4 protein expression in the RTN. Unlike other thalamic nuclei, the RTN is composed entirely of inhibitory projection and local-circuit neurons that express PV [57]. Thus, our current results indicate that ErbB4 expression occurs in several populations of PV-positive cells, both cerebral cortical interneurons and thalamic projection neurons.

ErbB4 receptor protein was also observed in the ventral mesencephalon of the rhesus monkey. However, our finding that ErbB4-immunoreactivity in dopaminergic nuclei is relatively lower than in the cortex was unexpected, because high levels of ErbB4 mRNA have been repeatedly reported in the substantia nigra and ventral tegmental area in both rodents and primates [9], [35], [37], [38], [53], [54]. Of note, we found that regions densely innervated by dopaminergic fibers displayed diffuse ErbB4 immunoreactivity (i.e., caudate, putamen, nucleus accumbens and additional regions of the ventral forebrain), raising the possibility that much of the protein within dopaminergic neurons may be transported to axons. This interpretation is supported by a recent study that demonstrated accumulation of the radioactively labeled NRG1-β1 extracellular domain in both the dorsal striatum and substantia nigra following systemic application in C57Bl/6 mice [58].

In addition, we detected high ErbB4 protein expression in the ventral hypothalamus of rhesus monkeys, which is consistent with previous studies in mice and humans that reported ErbB4 expression in hypothalamic astrocytes [41], [42]. Other investigations have implicated NRG1-ErbB4 signaling with the generation of oligodendrocytes in the midbrain and brain stem of different species [59], suggesting that ErbB4 expression may be less restricted to neurons in brain regions other than the telencephalon. Again, further studies are needed to characterize the exact cellular sources of hypothalamic ErbB4 during development and adulthood. In any case, the expression of the receptor protein in different cell types suggests that the NRG-ErbB4 pathway may well affect several functionally independent processes in the mammalian brain.

### Technical considerations

Our study was designed to provide a comprehensive qualitative analysis on ErbB4 expression throughout many cortical and subcortical areas of the monkey cerebrum. In order to provide this first extensive overview in primates, we had to restrict ourselves to qualitative approaches, relying mostly on assessment of the intensity of overall immunofluorescent signal in specific brain areas rather than on quantitative approaches such as cells counts. There are a few limitations that merit consideration when interpreting results using this type of qualitative approach. First, protein expression and fluorescence intensity may not be linear if there is steric interference of antibody binding and this limitation may be further affected when using an enzyme-based signal amplification procedure. Second, regional differences of tissue quality will influence any histological analysis; possible factors include variations in tissue thickness, uneven fixation as a function of the distance to blood vessels, as well as artifacts resulting from freezing, sectioning and tissue damage during the immunohistological processing. Third, inherent properties of the tissue can affect antibody penetration (i.e., highly myelinated areas) and the level of autofluorescence (i.e., lipofuscin-accumulation preferentially in some subpopulations of cells).

With respect to the extent of coexpression, or lack thereof, of two proteins in single cells, absence of co-immunolabeling is not sufficient proof to categorically determine that both proteins are either coexpressed or not in a cell population. This argument is pertinent to the absence of ErbB4 expression on cortical pyramidal cells that we identified with antibodies against neurogranin or neurofilament H in the present study, and additionally CaMKIIα in a recent publication [12]. However, because our immunohistological results were obtained using two different antibodies for ErbB4 and three independent markers of pyramidal cells, are consistent with prior *in situ* hybridization and single-cell PCR analyses in electrophysiologically identified cells in rodents [11], [12], we believe our conclusions regarding this point are well supported.

### Functional considerations

Our results show that the expression of ErbB4 protein is generally much higher in areas of the “corticolimbic system” compared to sensory-motor areas. We believe that this expression pattern is plausible with the genetic association of NRG1 and ErbB4 with schizophrenia, since the limbic association cortices are crucially involved in many of the functions affected in the disorder, e.g. selection of the focus of attention and suppression of irrelevant stimuli, working memory, selection and change of strategies [60], [61]. ErbB4 in the reticular thalamus is also of functional interest because this nucleus has been implicated in maintaining the focus of attention by selecting and enhancing relevant stimuli via afferents originating in the dorsolateral PFC and efferent inhibitory modulation of other thalamic nuclei [62]. The high expression levels in the amygdala and the nucleus accumbens implicate the NRG-ErbB4 signaling pathway in neuronal processes that are related to emotion, motivation, and reward [63]–[65]. Importantly, dense dopaminergic innervation from mesencephalic nuclei, especially the ventral tegmental area, is a common feature of limbic areas. The ventral tegmental area is directly innervated by projections from the prefrontal cortex (PFC) and is subject to modulation, or control, by the PFC. This pathway thus provides a feedback loop that allows the PFC to influence signal processing in other limbic areas indirectly via modulation of dopaminergic transmission [66], [67]. It has been shown that ErbB4 is not required for the development and maintenance of dopaminergic neurons [68]. However, application of NRG1 acutely augments dopamine release in the hippocampus [20] and protects mesencephalic dopaminergic neurons against 6-OHDA-induced neurotoxicity [69], suggesting that ErbB4 indeed modulates the function of dopaminergic neurons in different ways.

We conclude from our investigation that the large-scale organization of ErbB4 expression is largely conserved in rodents and primates. The results of our first assessment of ErbB4 protein expression throughout the cerebrum of primates thus validate the use of mice with genetically altered ErbB4 expression for investigations on the biological pathways and neuronal networks involved in the etiology of schizophrenia. Of note, our findings and data from other studies suggest that subcortical ErbB4 expression occurs in different populations of cells (dopaminergic and GABAergic projection neurons, astrocytes) and may also be targeted to axons; evidently, ErbB4 cellular and subcellular expression is less restricted in subcortical structures than in the cortex.

## Supporting Information

Figure S1 **Evaluation of area-specific ErbB4 immunofluorescent signal intensity.** Intensity of the green ErbB4 channel was evaluated in the entire area outlined by the purple line *(top)*. In addition, a histogram along the green line represents pixel intensities within each area *(bottom)*. Both the average pixel intensity throughout the entire areas as well as the line histograms indicate that immunofluorescent signal intensity is similarly high in the caudate-putamen compared to cortical area 23/24.
(TIF)

Figure S2 **Evaluation of relative differences of red and green channel intensities in cortical areas.** Images of cortical areas were smoothed with a Gaussian filter (Passes:1; Strength: 30 pixel) and the intensities of single color channels are presented throughout cortical layers (histograms along the yellow lines). Please note the differences between cortical areas with respect to relative signal intensities in the red and green channel. The red/green ratio is below 1 in area 46, about 1 in area V1, and above 1 in area F1, indicating an increase of immunoreactivity of the pyramidal cell marker neurofilament H (red channel) and a reduction of ErbB4-immunoreactivity (green channel) from limbic area 46 and sensory area V1 to motor cortex area F1, respectively.
(TIF)

Table S1 **Morphological definition of cortical and subcortical areas investigated.** Table S1 provides operational definitions for the location and boundaries of investigated brain areas. Abbreviations are the same as introduced throughout the text and in the figure legends.
(DOC)

## References

[1] Li D, Collier DA, He L (2006). Meta-analysis shows strong positive association of the neuregulin 1 (NRG1) gene with schizophrenia.. Hum Mol Genet.

[2] Silberberg G, Darvasi A, Pinkas-Kramarski R, Navon R (2006). The involvement of ErbB4 with schizophrenia: association and expression studies.. Am J Med Genet B Neuropsychiatr Genet.

[3] Buonanno A (2010). The neuregulin signaling pathway and schizophrenia: from genes to synapses and neural circuits.. Brain Res Bull.

[4] Georgieva L, Dimitrova A, Ivanov D, Nikolov I, Williams NM (2008). Support for neuregulin 1 as a susceptibility gene for bipolar disorder and schizophrenia.. Biol Psychiatry.

[5] Prata DP, Breen G, Osborne S, Munro J, St Clair D (2009). An association study of the neuregulin 1 gene, bipolar affective disorder and psychosis.. Psychiatr Genet.

[6] Nicodemus KK, Law AJ, Radulescu E, Luna A, Kolachana B (2010). Biological validation of increased schizophrenia risk with NRG1, ERBB4, and AKT1 epistasis via functional neuroimaging in healthy controls.. Arch Gen Psychiatry.

[7] Lai C, Lemke G (1991). An extended family of protein-tyrosine kinase genes differentially expressed in the vertebrate nervous system.. Neuron.

[8] Yau HJ, Wang HF, Lai C, Liu FC (2003). Neural development of the neuregulin receptor ErbB4 in the cerebral cortex and the hippocampus: preferential expression by interneurons tangentially migrating from the ganglionic eminences.. Cereb Cortex.

[9] Fox IJ, Kornblum HI (2005). Developmental profile of ErbB receptors in murine central nervous system: implications for functional interactions.. J Neurosci Res.

[10] Neddens J, Buonanno A (2010). Selective populations of hippocampal interneurons express ErbB4 and their number and distribution is altered in ErbB4 knockout mice.. Hippocampus.

[11] Vullhorst D, Neddens J, Karavanova I, Tricoire L, Petralia RS (2009). Selective expression of ErbB4 in interneurons, but not pyramidal cells, of the rodent hippocampus.. J Neurosci.

[12] Neddens J, Fish KN, Tricoire L, Vullhorst D, Shamir A (2011). ErbB4 is not detected in pyramidal cells but is confined to interneurons in the frontal cortex of humans, monkeys and rodents: Implications for schizophrenia.. Biol Psychiat.

[13] Wen L, Lu YS, Zhu XH, Li XM, Woo RS (2010). Neuregulin 1 regulates pyramidal neuron activity via ErbB4 in parvalbumin-positive interneurons.. Proc Natl Acad Sci U S A.

[14] Ting AK, Chen Y, Wen L, Yin DM, Shen C (2011). Neuregulin 1 promotes excitatory synapse development and function in GABAergic interneurons.. J Neurosci.

[15] Fazzari P, Paternain AV, Valiente M, Pla R, Lujan R (2010). Control of cortical GABA circuitry development by Nrg1 and ErbB4 signalling.. Nature.

[16] Huang YZ, Won S, Ali DW, Wang Q, Tanowitz M (2000). Regulation of neuregulin signaling by PSD-95 interacting with ErbB4 at CNS synapses.. Neuron.

[17] Kwon OB, Longart M, Vullhorst D, Hoffman DA, Buonanno A (2005). Neuregulin-1 reverses long-term potentiation at CA1 hippocampal synapses.. J Neurosci.

[18] Pitcher GM, Beggs S, Woo RS, Mei L, Salter MW (2008). ErbB4 is a suppressor of long-term potentiation in the adult hippocampus.. Neuroreport.

[19] Chen YJ, Zhang M, Yin DM, Wen L, Ting A (2010). ErbB4 in parvalbumin-positive interneurons is critical for neuregulin 1 regulation of long-term potentiation.. Proc Natl Acad Sci U S A.

[20] Kwon OB, Paredes D, Gonzalez CM, Neddens J, Hernandez L (2008). Neuregulin-1 regulates LTP at CA1 hippocampal synapses through activation of dopamine D4 receptors.. Proc Natl Acad Sci U S A.

[21] Fisahn A, Neddens J, Yan L, Buonanno A (2009). Neuregulin-1 Modulates Hippocampal Gamma Oscillations: Implications for Schizophrenia.. Cereb Cortex.

[22] Anton ES, Ghashghaei HT, Weber JL, McCann C, Fischer TM (2004). Receptor tyrosine kinase ErbB4 modulates neuroblast migration and placement in the adult forebrain.. Nat Neurosci.

[23] Flames N, Long JE, Garratt AN, Fischer TM, Gassmann M (2004). Short- and long-range attraction of cortical GABAergic interneurons by neuregulin-1.. Neuron.

[24] Krivosheya D, Tapia L, Levinson JN, Huang K, Kang Y (2008). ErbB4-Neuregulin signaling modulates synapse development and dendritic arborization through distinct mechanisms.. J Biol Chem.

[25] Lein ES, Hawrylycz MJ, Ao N, Ayres M, Bensinger A (2007). Genome-wide atlas of gene expression in the adult mouse brain.. Nature.

[26] Thompson M, Lauderdale S, Webster MJ, Chong VZ, McClintock B (2007). Widespread expression of ErbB2, ErbB3 and ErbB4 in non-human primate brain.. Brain Res.

[27] Hahn CG, Wang HY, Cho DS, Talbot K, Gur RE (2006). Altered neuregulin 1-erbB4 signaling contributes to NMDA receptor hypofunction in schizophrenia.. Nat Med.

[28] Bernstein HG, Lendeckel U, Bertram I, Bukowska A, Kanakis D (2006). Localization of neuregulin-1alpha (heregulin-alpha) and one of its receptors, ErbB-4 tyrosine kinase, in developing and adult human brain.. Brain Res Bull.

[29] Chen X, Levkowitz G, Tzahar E, Karunagaran D, Lavi S (1996). An immunological approach reveals biological differences between the two NDF/heregulin receptors, ErbB-3 and ErbB-4.. J Biol Chem.

[30] Singec I, Knoth R, Ditter M, Volk B, Frotscher M (2004). Neurogranin is expressed by principal cells but not interneurons in the rodent and monkey neocortex and hippocampus.. J Comp Neurol.

[31] Higo N, Oishi T, Yamashita A, Murata Y, Matsuda K (2007). Expression of protein kinase-C substrate mRNA in the motor cortex of adult and infant macaque monkeys.. Brain Res.

[32] Campbell MJ, Morrison JH (1989). Monoclonal antibody to neurofilament protein (SMI-32) labels a subpopulation of pyramidal neurons in the human and monkey neocortex.. J Comp Neurol.

[33] Jinno S, Aika Y, Fukuda T, Kosaka T (1998). Quantitative analysis of GABAergic neurons in the mouse hippocampus, with optical disector using confocal laser scanning microscope.. Brain Res.

[34] Saleem KS, Logothetis NK (2007). A Combined MRI and Histology Atlas of the Rhesus Monkey Brain.. Academic Press; London, UK.

[35] Steiner H, Blum M, Kitai ST, Fedi P (1999). Differential expression of ErbB3 and ErbB4 neuregulin receptors in dopamine neurons and forebrain areas of the adult rat.. Exp Neurol.

[36] Chen YJ, Johnson MA, Lieberman MD, Goodchild RE, Schobel S (2008). Type III neuregulin-1 is required for normal sensorimotor gating, memory-related behaviors, and corticostriatal circuit components.. J Neurosci.

[37] Woo RS, Li XM, Tao Y, Carpenter-Hyland E, Huang YZ (2007). Neuregulin-1 Enhances Depolarization-Induced GABA Release.. Neuron.

[38] Gerecke KM, Wyss JM, Karavanova I, Buonanno A, Carroll SL (2001). ErbB transmembrane tyrosine kinase receptors are differentially expressed throughout the adult rat central nervous system.. J Comp Neurol.

[39] Hou J, Li B, Yang Z, Fager N, Ma MY (2002). Functional integrity of ErbB-4/-2 tyrosine kinase receptor complex in the hypothalamus is required for maintaining normal reproduction in young adult female rats.. Endocrinology.

[40] Ma YJ, Hill DF, Creswick KE, Costa ME, Cornea A (1999). Neuregulins signaling via a glial erbB-2-erbB-4 receptor complex contribute to the neuroendocrine control of mammalian sexual development.. J Neurosci.

[41] Prevot V, Rio C, Cho GJ, Lomniczi A, Heger S (2003). Normal female sexual development requires neuregulin-erbB receptor signaling in hypothalamic astrocytes.. J Neurosci.

[42] Sharif A, Duhem-Tonnelle V, Allet C, Baroncini M, Loyens A (2009). Differential erbB signaling in astrocytes from the cerebral cortex and the hypothalamus of the human brain.. Glia.

[43] Knight RT, Nakada T (1998). Cortico-limbic circuits and novelty: a review of EEG and blood flow data.. Rev Neurosci.

[44] Ganzel BL, Morris PA, Wethington E (2010). Allostasis and the human brain: Integrating models of stress from the social and life sciences.. Psychol Rev.

[45] Elman I, Borsook D, Lukas SE (2006). Food intake and reward mechanisms in patients with schizophrenia: implications for metabolic disturbances and treatment with second-generation antipsychotic agents.. Neuropsychopharmacology.

[46] Benes FM (2010). Amygdalocortical circuitry in schizophrenia: from circuits to molecules.. Neuropsychopharmacology.

[47] Coyle JT, Balu D, Benneyworth M, Basu A, Roseman A (2010). Beyond the dopamine receptor: novel therapeutic targets for treating schizophrenia.. Dialogues Clin Neurosci.

[48] Swerdlow NR (2010). Integrative circuit models and their implications for the pathophysiologies and treatments of the schizophrenias.. Curr Top Behav Neurosci.

[49] McMahon LL, Williams JH, Kauer JA (1998). Functionally distinct groups of interneurons identified during rhythmic carbachol oscillations in hippocampus in vitro.. J Neurosci.

[50] Soriano E, Frotscher M (1993). Spiny nonpyramidal neurons in the CA3 region of the rat hippocampus are glutamate-like immunoreactive and receive convergent mossy fiber input.. J Comp Neurol.

[51] Arabadzisz D, Freund TF (1999). Changes in excitatory and inhibitory circuits of the rat hippocampus 12-14 months after complete forebrain ischemia.. Neuroscience.

[52] Ghashghaei HT, Weber J, Pevny L, Schmid R, Schwab MH (2006). The role of neuregulin-ErbB4 interactions on the proliferation and organization of cells in the subventricular zone.. Proc Natl Acad Sci U S A.

[53] Meyer D, Yamaai T, Garratt A, Riethmacher-Sonnenberg E, Kane D (1997). Isoform-specific expression and function of neuregulin.. Development.

[54] Pinkas-Kramarski R, Eilam R, Alroy I, Levkowitz G, Lonai P (1997). Differential expression of NDF/neuregulin receptors ErbB-3 and ErbB-4 and involvement in inhibition of neuronal differentiation.. Oncogene.

[55] Dickerson JW, Hemmerle AM, Numan S, Lundgren KH, Seroogy KB (2009). Decreased expression of ErbB4 and tyrosine hydroxylase mRNA and protein in the ventral midbrain of aged rats.. Neuroscience.

[56] Disney AA, Aoki C (2008). Muscarinic acetylcholine receptors in macaque V1 are most frequently expressed by parvalbumin-immunoreactive neurons.. J Comp Neurol.

[57] Csillik B, Mihaly A, Krisztin-Peva B, Chadaide Z, Samsam M (2005). GABAergic parvalbumin-immunoreactive large calyciform presynaptic complexes in the reticular nucleus of the rat thalamus.. J Chem Neuroanat.

[58] Rosler TW, Depboylu C, Arias-Carrion O, Wozny W, Carlsson T (2011). Biodistribution and brain permeability of the extracellular domain of neuregulin-1-beta1.. Neuropharmacology [Epub ahead of print].

[59] Wood JD, Bonath F, Kumar S, Ross CA, Cunliffe VT (2009). Disrupted-in-schizophrenia 1 and neuregulin 1 are required for the specification of oligodendrocytes and neurones in the zebrafish brain.. Hum Mol Genet.

[60] Goto Y, Yang CR, Otani S (2010). Functional and dysfunctional synaptic plasticity in prefrontal cortex: roles in psychiatric disorders.. Biol Psychiatry.

[61] Kalkstein S, Hurford I, Gur RC (2010). Neurocognition in schizophrenia.. Curr Top Behav Neurosci.

[62] Zikopoulos B, Barbas H (2006). Prefrontal projections to the thalamic reticular nucleus form a unique circuit for attentional mechanisms.. J Neurosci.

[63] Faure A, Reynolds SM, Richard JM, Berridge KC (2008). Mesolimbic dopamine in desire and dread: enabling motivation to be generated by localized glutamate disruptions in nucleus accumbens.. J Neurosci.

[64] Burns LH, Annett L, Kelley AE, Everitt BJ, Robbins TW (1996). Effects of lesions to amygdala, ventral subiculum, medial prefrontal cortex, and nucleus accumbens on the reaction to novelty: implication for limbic-striatal interactions.. Behav Neurosci.

[65] Morrison SE, Salzman CD (2010). Re-valuing the amygdala.. Curr Opin Neurobiol.

[66] Sesack SR, Carr DB, Omelchenko N, Pinto A (2003). Anatomical substrates for glutamate-dopamine interactions: evidence for specificity of connections and extrasynaptic actions.. Ann N Y Acad Sci.

[67] Laviolette SR (2007). Dopamine modulation of emotional processing in cortical and subcortical neural circuits: evidence for a final common pathway in schizophrenia?. Schizophr Bull.

[68] Thuret S, Alavian KN, Gassmann M, Lloyd CK, Smits SM (2004). The neuregulin receptor, ErbB4, is not required for normal development and adult maintenance of the substantia nigra pars compacta.. J Neurochem.

[69] Zhang L, Fletcher-Turner A, Marchionni MA, Apparsundaram S, Lundgren KH (2004). Neurotrophic and neuroprotective effects of the neuregulin glial growth factor-2 on dopaminergic neurons in rat primary midbrain cultures.. J Neurochem.

