# Supplementary figures and images for: Expression of the Neuregulin Receptor ErbB4 in the Brain of the Rhesus Monkey (Macaca mulatta)
Source: PLoS One. 2011 Nov 8;6(11):e27337. doi: 10.1371/journal.pone.0027337 (PMC3210802; doi:10.1371/journal.pone.0027337)

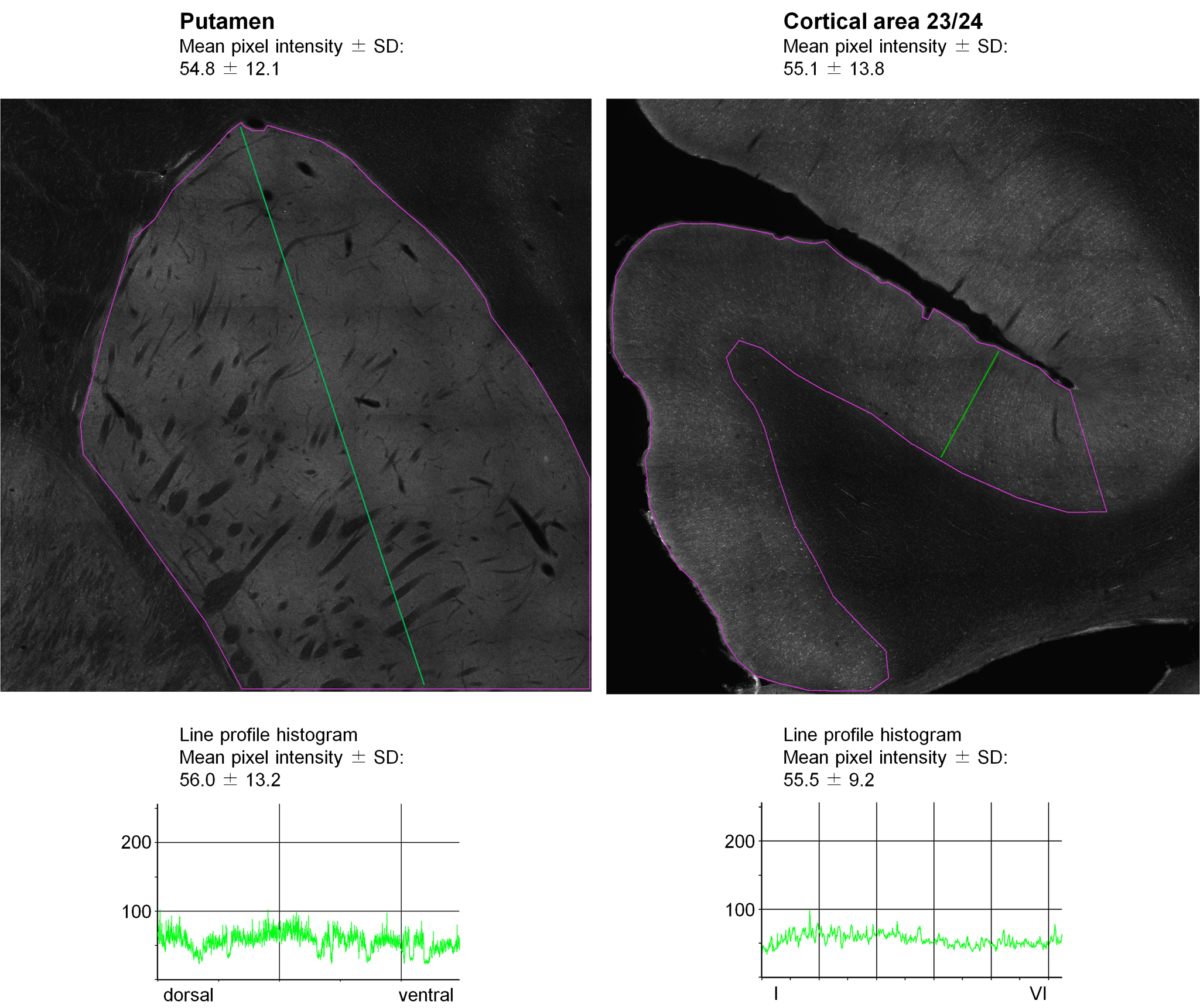

Supplement: Figure S1 — Evaluation of area-specific ErbB4 immunofluorescent signal intensity. Intensity of the green ErbB4 channel was evaluated in the entire area outlined by the purple line (top). In addition, a histogram along the green line represents pixel intensities within each area (bottom). Both the average pixel intensity throughout the entire areas as well as the line histograms indicate that immunofluorescent signal intensity is similarly high in the caudate-putamen compared to cortical area 23/24. (TIF) [file pone.0027337.s001.tif]

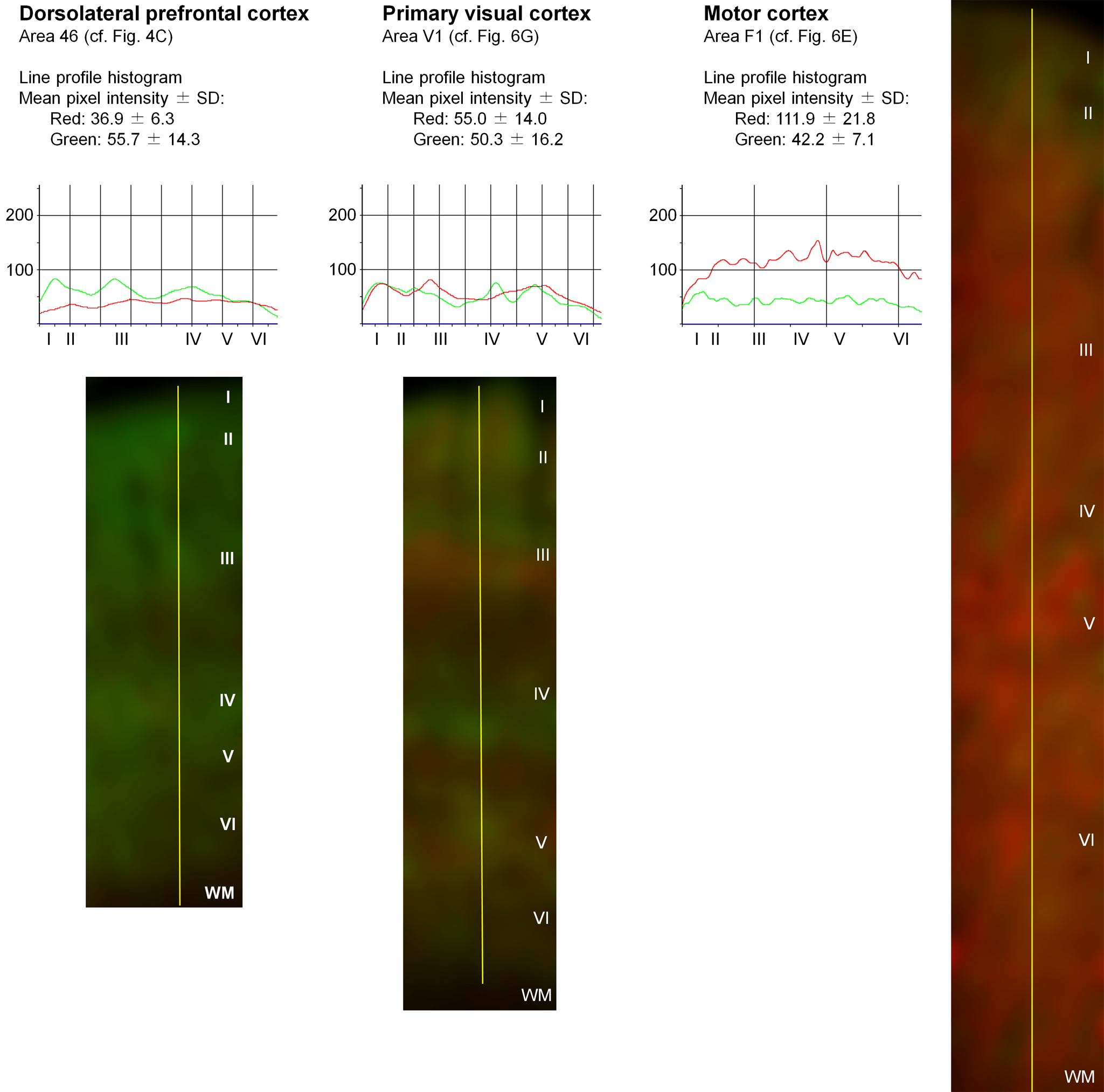

Supplement: Figure S2 — Evaluation of relative differences of red and green channel intensities in cortical areas. Images of cortical areas were smoothed with a Gaussian filter (Passes:1; Strength: 30 pixel) and the intensities of single color channels are presented throughout cortical layers (histograms along the yellow lines). Please note the differences between cortical areas with respect to relative signal intensities in the red and green channel. The red/green ratio is below 1 in area 46, about 1 in area V1, and above 1 in area F1, indicating an increase of immunoreactivity of the pyramidal cell marker neurofilament H (red channel) and a reduction of ErbB4-immunoreactivity (green channel) from limbic area 46 and sensory area V1 to motor cortex area F1, respectively. (TIF) [file pone.0027337.s002.tif]
