# Supplementary material for: Expression of the Neuregulin Receptor ErbB4 in the Brain of the Rhesus Monkey (Macaca mulatta)
Source: PLoS One. 2011 Nov 8;6(11):e27337. doi: 10.1371/journal.pone.0027337 (PMC3210802; doi:10.1371/journal.pone.0027337)
Supplement: Table S1 — Morphological definition of cortical and subcortical areas investigated. Table S1 provides operational definitions for the location and boundaries of investigated brain areas. Abbreviations are the same as introduced throughout the text and in the figure legends. (DOC) [file pone.0027337.s003.doc]

| **Area** | **Operational definition of areas** |
| --- | --- |
| InC | Cortical area between the superior and inferior limbs of the limiting sulcus |
| S2 | Cortical area adjacent to InC dorsolateral to the superior limb of the limiting sulcus; intensity measurement in a 500µm-wide radial profile |
| 23/24 | Cortical area extending from ventricle (medial) to the lateral limb of the limiting sulcus |
| 25 | Cortical area medial of the Cd and NAC on subgenual or nearby pregenual sections |
| 32 | Cortical area ventral of the cingulate sulcus on prefrontal sections |
| 46 | Cortical area within the principal sulcus on prefrontal sections |
| F1 | Occurrence of giant Betz cell somata in layer V |
| F3 | Occurrence of very large pyramidal somata in layer V; intensity measurement in a 500µm-wide radial profile |
| S1-2 | Cortical area on the dorsolateral surface between two sulci; intensity measurement in a 500µm-wide radial profile |
| V1 | Occurrence of macroscopic striate morphology in layer IV |
| NAC | Area ventral of Cd/Pu on pregenual sections, separated from Cd/Pu by drawing a line from the ventral tip of the lateral ventricle parallel to the ventral brain surface |
| Cd | Dorsal striatal nucleus highlighted by TH-immunofluorescence, the small Cd cauda may lie ventral to Pu |
| Pu | Ventral striatal nucleus highlighted by TH-immunofluorescence, separated from Cd by the internal capsule |
| Cl | TH-negative area surrounded by white matter between Pu and InC |
| GPe | Largely TH-negative area surrounded by white matter medial of Pu |
| GPi | Largely TH-negative area surrounded by white matter medial of GPe |
| AMY | Large area ventral of the optic tract and GPe/i, extending from medial surface to lateral white matter, ventral boundary by white matter between AMY and ECi; individual nuclei were identified using information from the monkey brain atlas and primary research publications |
| RTN | Large elongated and bent nucleus highlighted by strong PV-immunofluorescence |
| SN | Elongated area with strongly TH-immunofluorescent somata in brainstem medial from optic tract |
| VTA | Round to triangular area with strongly TH-immunofluorescent somata close to midline in brainstem ventromedial from SN |
